# A randomized controlled trial comparing isosorbide dinitrate-oxytocin versus misoprostol-oxytocin at management of foetal intrauterine death

**DOI:** 10.1371/journal.pone.0215718

**Published:** 2019-11-21

**Authors:** Gabriel Arteaga-Troncoso, Aide E. Chacon-Calderon, Francisco J. Martinez-Herrera, Sylvia G. Cruz-Nuñez, Marcela Lopez-Hurtado, Aurora Belmont-Gomez, Alberto M. Guzman-Grenfell, Blanca E. Farfan-Labonne, Carlos J. Neri-Méndez, Francisco Zea-Prado, Fernando M. Guerra-Infante

**Affiliations:** 1 Department of Cellular Biology and Development, National Institute of Perinatology, Mexico City, Mexico; 2 Department of Obstetrics and Gynecology, Women’s Clinic and Neonatology, Secretariat for National Defense, Mexico City, Mexico; 3 Department of Obstetric and Gynecology, National Institute of Perinatology, Mexico City, Mexico; 4 Department of Infectology and Immunology, National Institute of Perinatology, Mexico City, Mexico; 5 Department of Clinical Pharmacology, National Institute of Perinatology, Mexico City, Mexico; 6 Department of Immunobiochemistry, National Institute of Perinatology, Mexico City, Mexico; 7 Department of Neurosciences, National Institute of Perinatology, Mexico City, Mexico; Stavanger University Hospital, NORWAY

## Abstract

**Background:**

The metabolic activity of endogenous nitric oxide (NO) and the medical use of nitrovasodilatory drugs like isosorbide dinitrate have been shown to be potential inducers inducers of cervical ripening prior to surgical evacuation of the uterus.

**Objective:**

To assess the therapeutic efficacy and safety of combined isosorbide dinitrate-oxytocin in the management of intrauterine foetal death (IUFD).

**Methods:**

Sixty women with IUFD after 20 weeks of gestation requesting uterine evacuation were randomly selected to receive isosorbide dinitrate gel solution (80 mg/1.5 mL; n = 30) or misoprostol gel solution (100 mcg/1.5 mL; n = 30) every 3 h with a maximum of four doses or until a Bishop score >7 was reached. Subsequently, patients received a high dose of intravenous oxytocin until complete uterus evacuation was achieved. Therapeutic efficacy was evaluated by mean the relative risk of the foetal expulsion based on comparison of event rates, and the proportion of women induced to labor at 7, 10 and 15 h after the administration of isosorbide dinitrate or misoprostol. Safety was assessed on the basis of woman´s vital signs and evaluation of adverse effects, including headache, abdominal pain, pelvic pain, lower back pain, nausea, dizziness and vomiting.

**Results:**

The foetal expulsion rate using the isosorbide dinitrate-oxytocin combination was approximately 4.4 times, and at least 2.1 times, the foetal expulsion rate with the misoprostol-oxytocin regimen at any given point in time. The proportion of women achieved vaginal delivery at 15 hours was 100% for the isosorbide dinitrate-oxytocin group and 86.7% for the misoprostol-oxytocin group. The average delivery induction interval was significantly lower when isosorbide dinitrate-oxytocin was used (8.7 ± 3.1 h) than when misoprostol-oxytocin (11.9 ± 3.1 h) was used. A total of 20% of patients in the isosorbide dinitrate-oxytocin group recorded headache, and no cases of uterine tachysystole, haemorrhage or coagulopathy were recorded.

**Conclusion:**

This study indicates that intravaginal isosorbide dinitrate followed by intravenous oxytocin was more effective than the conventional method used to induce labour in the medical management of foetal death in pregnancies after 20 weeks of gestation.

**Trial registration:**

Clinicaltrials.gov NCT02488642.

## Introduction

Intrauterine foetal death (IUFD) is one of the most devastating episodes in obstetrics during which the mother, father and family suffer an intense emotional crisis. A long delay in labour can increase the risk of anxiety and psychological pain related to long-term symptoms and the probability of a caesarean delivery. Women with little social support are particularly vulnerable to anxiety and postpartum depression [1,2].

After the diagnosis of IUFD, most hospitalised patients opt to deliver the baby within 48 h [3]. The induction of labour in a pregnant woman with an unripe cervical opening can lead to a failed induction and, invariably, birth by caesarean section. The pharmacological induction of labour is an obstetric procedure that artificially initiates uterine concentrations that lead to progressive dilation and cervical ripening prior to the administration of intravenous oxytocin [4]. For induction in cases of IUFD, methods of cervical preparation include the vaginal administration of dinoprostone, gemeprost, misoprostol, oxytocin and progesterone receptor antagonists [5,6]. The main problems reported by women during the induction of labour are excessive uterine activity and ineffective labour, which, when associated with a history of placenta praevia or uterine surgery, can increase the risk of uterine rupture and bleeding [7,8].

The therapeutic efficacy and safety of nitric oxide (NO) donor drugs have been reported, and isosorbide dinitrate has been used to induce cervical ripening during the first trimester of gestation [9]. Organic nitrates are reductively metabolised to release NO, which stimulates the activation of soluble guanylyl cyclase (GC). This activation leads to the conversion of guanosine triphosphate (GTP) into cyclic guanosine monophosphate (cGMP), the second messenger involved in NO mediation. The increased cGMP level causes the smooth musculature to relax [10–13]. In addition, organic nitrates participate in the endogenous NO production pathway in endothelial cells to maintain vascular tone [14–16]. This mechanism has generated interest in exploring the metabolic activity of NO and the medical use of nitrovasodilatory drugs as potential inducers of cervical ripening. The objective of this study was to compare the efficacy and adverse effects of the intracervical administration of isosorbide dinitrate gel followed by a high dose of oxytocin with those of a commonly utilised combined regimen—misoprostol-oxytocin—for the induction of labour after IUFD.

## Materials and methods

The study was approved by the Research Ethics Committee of the National Institute of Perinatology Isidro Espinosa de los Reyes (CONBIOETICA-09-CEI-021-20170823. Protocol number: 212250–29021, date of issue: November 2008) and was registered in ClinicalTrials.gov (registration number NCT02488642). The research was conducted according to the principles expressed in the Declaration of Helsinki, and all women participants were adequately informed about the study objectives stating their consent to participate by signing written informed consent documents.

The study was performed in the obstetrics department of the “Isidro Espinosa de los Reyes” National Institute of Perinatology, Department of Health, and the Women’s Clinic at the Secretariat for National Defense. A pilot phase was conducted to test the procedures for patient recruitment and data collection and provide estimates of event rates for the sample size calculation. The first patient was enrolled in May 2009, and date that last participant was received an intervention for the purposes of final collection of data for the primary outcome at September 2013. A complete statistical analysis, including only cases the patients that met the inclusion criteria for which clinical efficacy and safety data were available, was undertaken. There was no imputation for missing data, and unblinded interim analyses were provided to the Research Ethics Committee that manifested the actual study completion date in May 2014.

A prospective, randomised, double-blind, controlled clinical trial was conducted to compare the efficacy and clinical safety of the induction of labour using the combination of isosorbide dinitrate-oxytocin (experimental arm, X = 1) and misoprostol-oxytocin (standard arm, X = 0). Main outcome measures were the relative risk of foetal expulsion based on comparison of event rates between treatment groups, and the proportion of women delivering within 7, 10 and 15 h after the administration of isosorbide dinitrate or misoprostol.

**Fig 1:**
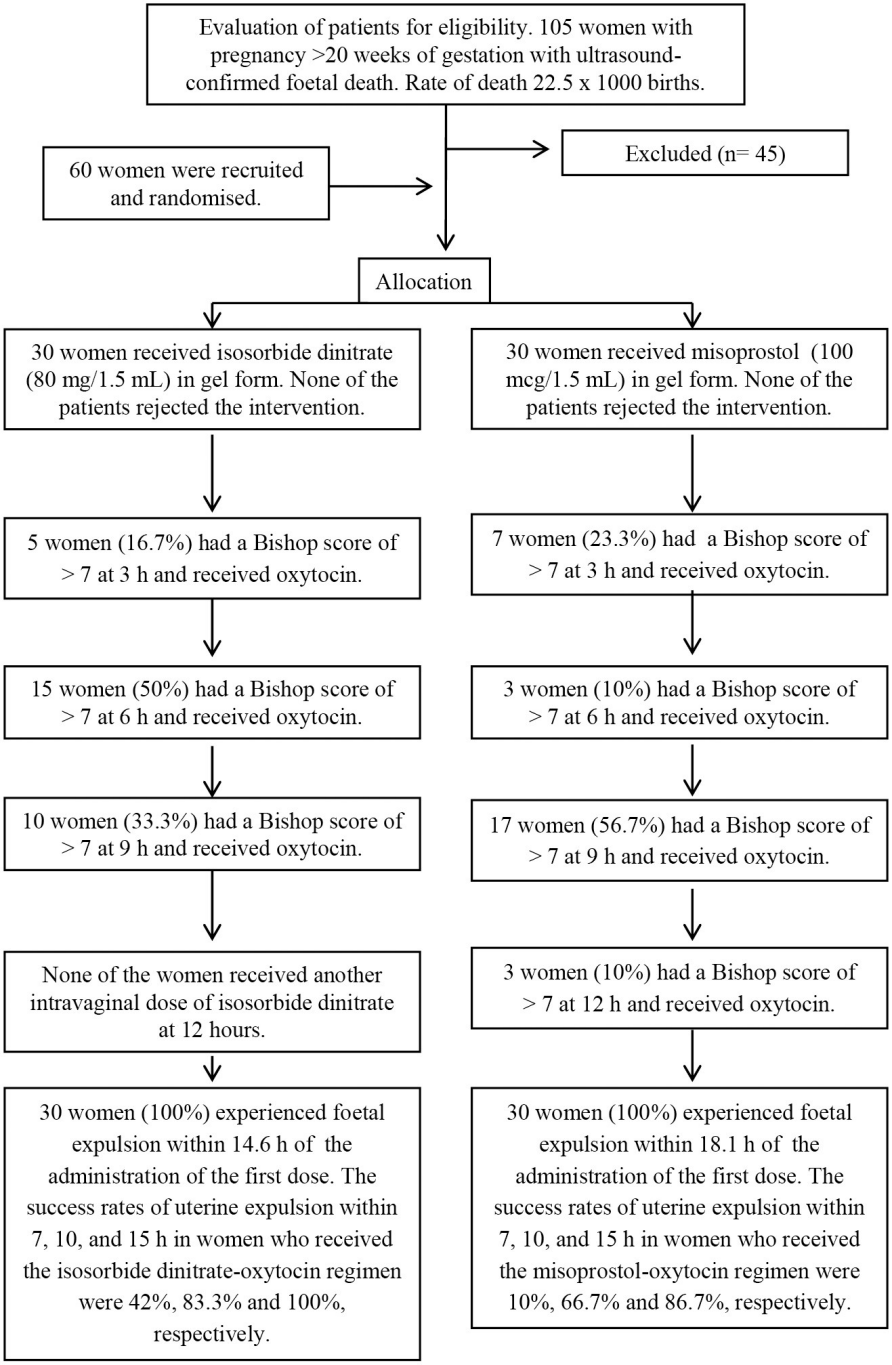
CONSORT flow diagram. After attaining a Bishop score of > 7, the women received no additional doses of isosorbide dinitrate or misoprostol and were given an infusion of oxytocin in a balanced electrolyte solution beginning with an infusion rate of 2 mUI/min and increasing in 15-min intervals.

In total, 60 women with pregnancies exceeding 20 weeks gestation were referred for foetal evacuation (Fig 1; S1 CONSORT checklist and S1 Protocol). We defined late IUFD as foetuses without signs of life in the uterus after 20 complete weeks of pregnancy. Women with the following characteristics were included: closed cervix without evidence of cervical dilation or baseline uterine activity, a Bishop score of <5, intact membranes, gestation greater than or equal to 20 weeks established by the date of menstruation or by foetometry, and ultrasound-confirmed late IUFD. This study did not include the medical management of multiple pregnancies, IUFD after late foeticide, specific medical conditions associated with an increased risk of IUFD, patients with a history of hypertension, or women with a history of unexplained antepartum haemorrhage, pelvic dystocia or any another counter-indications where medications were used.

Unrestricted randomization based on a simple sequence determined the allocation of each subject to the treatment group and patients were assigned to receive isosorbide dinitrate or misoprostol. Group allocation was predetermined and randomization could be achieved simply by registering each new recruit by assigning them the number in the product package in numerical order. A single person who was blinded to the treatment allocation examined all participants vaginally. The medications were administered in the posterior fornix, and cervical activity was evaluated at baseline and every 3 h to monitor any change based on the Bishop score.

If cervical conditions did not change after treatment application, the participants received another dose (the maximum number of doses was four) to facilitate cervical ripening. Independent of the treatment received, once a Bishop score >7 was achieved, oxytocin was infused in a balanced electrolyte solution; the initial infusion rate was 2 mIU/min, and the dose doubled every 15 min. Labour induction time from first administration of medication to expulsion of the foetus was determined with a digital stopwatch. Prior to the administration of each subsequent dose, each woman's vital signs were verified to confirm that she was in stable condition and demonstrated haemodynamic stability. During this time, data and medical information were collected on paper and then entered into a computational database. Participants remained at rest during the evaluation of adverse effects, including headache, abdominal pain, pelvic pain, lower back pain, nausea, dizziness and vomiting. A lack of cervical activity after four doses was considered treatment failure.

An isosorbide dinitrate or misoprostol-based solution was prepared according to previously published specifications [9]. Chemical and pharmacological details appear as support in S1 Materials and Methods. The reagents had final concentrations of 80 mg of isosorbide dinitrate or 100 mcg of misoprostol in 1.5 mL of gel solution. Both solutions had the same appearance and were packed in similar syringes to keep physicians, patients and researchers blinded regarding the treatment. The pharmacist was the only individual who knew the contents of the syringes. Four syringes were placed in an opaque and sealed envelope consecutively numbered with a unique study number. The physician administered the contents of the syringe, repeating this process every 3 h, according to the prior selection and random allocation of patients. The entire "stock" of reagents remained stable for a period of 20 d and was maintained at room temperature (18–25°C) before use.

The sample size was calculated using the Cox regression model with the risk quotient (log hazard ratio) and 95% confidence intervals (95% CI) [17]. Sixty patients (30 in each arm) were required to ensure 80% power to detect a 50% risk reduction in the experimental group relative to the control group (log hazard-ratio of ln(0.5) = ‒0.17) with a one-sided test and an α value of 0.05.

All continuous variables were summarised using histograms and central tendency measures before conducting Chi-square, Fisher’s exact, Mantel-Haenszel, and Student’s t-tests, as appropriate. The Shapiro-Wilk test was used to verify the normality of the data, and we applied Cox regression to model the time-event of labour induction after IUFD, controlling for maternal age, foetal size and weight and weeks of gestation as covariates. The proportional hazards assumption was evaluated using the two graphed lines, which corresponded to the survival curves (absence of crossing) and the log-log plots. Systolic and diastolic blood pressures, heart and respiratory rates, as well as temperature were recorded at baseline and subsequently every 3 h before the application of a new dose of isosorbide dinitrate or misoprostol. This data was performed by analysis of variance with a general linear model of repeated measures and the difference between groups at each level of each factor. Interactions were considered significant if p-values for the interaction term were not greater than 0.05 (Bonferroni correction). Sphericity violation was corrected using Greenhouse-Geisser adjustment to the number of degrees of freedom. Post hoc independent samples t-tests were performed to identify vital signs showing significant differences between groups as follow-ups to significant interaction effects, and were considered significant if p-values were not greater than 0.05. Relative risks (RRs) and 95% CIs were estimated utilising bivariate analysis, including non-serious adverse events among the treatment groups. Attributable risk was calculated to determine the probability of the total risk of each adverse effect observed with the use of isosorbide dinitrate or misoprostol. All of the tests were two-tailed, and results with p-values < 0.05 were considered statistically significant. Stata/IC v.13.0 (Stata Corp LP, College Station, Texas, USA) was utilised to perform the statistical analyses.

## Results

A summary of the patient cohort and their participation in the trial is shown in Fig 1.

Of the 105 potential candidates women who were screened, 60 women with IUFD were enrolled and treated with isosorbide dinitrate-oxytocin or misoprostol-oxytocin. Although there was some variability at birthweight, baseline patient characteristics and obstetric parameters were generally similar across treatment groups (Table 1).

**Table 1:** Patient characteristics and obstetric parameters.

Characteristic	Combined regimen	
	Isosorbide dinitrate-oxytocin	Misoprostol-oxytocin
Age (years)	27.4 (±8.1)	27.9 (±5.7)
Parity	1 [0–1]	1 [0–3]
Gestation at Induction (weeks)	28.1 (±5.9)	27.6 (±6)
Birthweight (g)*	1251.5 (±707.2)	717 (±381.3)
Height (cm)	36.9 (±8.5)	32.4 (5.7)

Continuous variables are presented as mean [SD] and median [range].

* P-value < .003 by Student´s t test.

**Fig 2:**
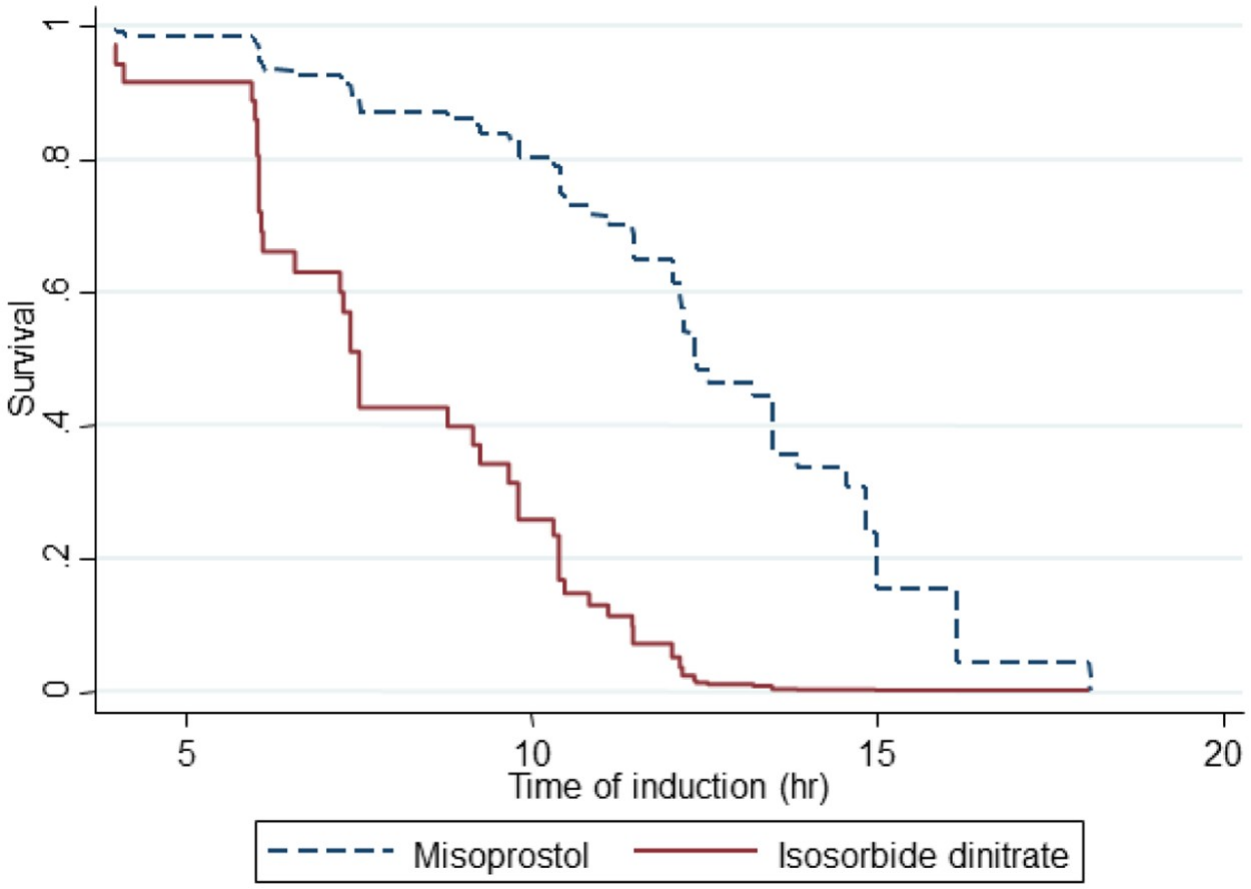
Kaplan-Meier curve illustrating overall survival based on treatments. The risk of remaining in the study reflected the interval of time between the administration of the first dose of isosorbide dinitrate or misoprostol followed by oxytocin until the foetal expulsion event.

The probability of the risk of remaining in the study reflected the time interval between the administration of the first dose of isosorbide dinitrate or misoprostol followed by oxytocin and the foetal expulsion event. We observed evidence of a pharmacological effect in the groups, which varied linearly with expulsion time (Fig 2).

The high risk ratio (HR 4.4, 95% CI 2.1–9, p <0.001) indicated that treatment with isosorbide dinitrate-oxytocin helped patients to progress with foetal expulsion much better than those who received misoprostol-oxytocin at any given point in time. This relationship also indicated to what extent treatment with isosorbide dinitrate-oxytocin can shorten the duration of foetal expulsion.

The success rates of uterine expulsion within 7, 10 and 15 h in the isosorbide dinitrate-oxytocin group were 42%, 83.3% and 100%, respectively. In contrast, the successful expulsion rates in women who received the misoprostol-oxytocin regimen were 10%, 66.7% and 86.7%, respectively, at the same times. Four women experienced foetal expulsion within 18.1 h of initiating the misoprostol-oxytocin regimen. The average administration induction intervals were significantly lower (p < 0.001) using the combined isosorbide dinitrate-oxytocin (8.7 ± 3.1 h) versus the misoprostol-oxytocin regimen (11.9 ± 3.1 h). Although we found no significant correlation between the time of expulsion and maternal age, foetal size or weight, the expulsion time interval increased as the weeks of gestation increased (r = 0.31; p < 0.02).

Of the 30 women who received isosorbide dinitrate, 16.7% reached a Bishop score of > 7 after one dose and thus received oxytocin, 50% reached a score of >7 after two applications, and 33.3% after three applications. In contrast, this value was reached by 23.3% of the 30 women after one misoprostol application, 10% after two applications, 56.7% after three applications and 10% after four applications. Women in the isosorbide dinitrate group required significantly fewer doses than women in the misoprostol group (p < 0.001; Mantel-Haenszel test).

There was a slight decrease in systolic and diastolic blood pressure with an increase in heart rate after the administration of 80 mg of isosorbide dinitrate in gel solution (p <0.001; Greenhouse-Geisser adjustment) (Table 2). The reduction in mean systolic blood pressure between isosorbide dinitrate and misoprostol after the first two doses was slightly lower, with a difference of 6.7 mm Hg (95% confidence interval of the difference, 5.6–7.7 mm Hg; p < 0.001) and 7.6 mm Hg (95% confidence interval of the difference, 6.5–8.7 mm Hg; p < 0.001), respectively. The isosorbide dinitrate also experienced a slight decrease in mean diastolic blood pressure, with a difference of 4.6 mm Hg (95% confidence interval of the difference, 2.7–6.6 mm Hg; p < 0.001) and 6.1 mm Hg (95% confidence interval of the difference, 4.3–7.9 mm Hg; p < 0.001), respectively. There was increase in heart rate after the administration of 80 mg isosorbide dinitrate. The difference in mean heart rate between isosorbide dinitrate and misoprostol was higher after two doses, with a difference of 9.5 beats/min (95% confidence interval, 6.7–12.2 beats/min). There was no significant difference in respiratory frequency between isosorbide dinitrate and misoprostol groups, and increase of 0.1–0.6°C in core temperature after the first dose of prostaglandin treatment (p < 0.001).

**Table 2:** Blood pressures, heart and respiratory rates, and core temperature prior to the administration of subsequent dose of isosorbide dinitrate or misoprostol. Mean (Std. Deviation).

	Doses (number)				
	Baseline	1	2	3	4
**Isosorbide dinitrate-oxytocin regimen**					
No. of women who had Bishop´s score >7^&^	30	5	15	10	0
Heart rate (bpm)	79.3 (4.5)	81.9 (3.6)***	89.6 (5.2)***	89.4 (6.9)***	85.9 (7.8)***
Core temperature (°C)	36.6 (0.3)	36.6 (0.1)	36.9 (0.5)	36.9 (0.4)	37.2 (0.4)
Systolic blood pressure (mmHg)	120.1 (1.7)	112 (2.9)***	110.7 (1.4)***	113.9 (4.1)***	118.1 (3.7)*
Diastolic blood pressure (mmHg)	74 (4.4)	72 (3.5)***	70.7 (2.8)***	72.8 (3.8)***	74.4 (4.3)
Respiratory frequency (minutes)	21.3 (1.6)	21.6 (1.8)	21.7 (2.2)	21.6 (1.3)	21.2 (1.4)
**Misoprostol-oxytocin regimen**					
No. of women who had Bishop´s score >7^&^	30	7	3	17	3
Heart rate (bpm)	79 (5.3)	78.4 (4.4)	80.1 (5.4)	80.5 (4.8)	79.9 (4)
Core temperature (°C)	36.6 (0.5)	36.7 (0.2)**	37.4 (0.6)**	37.4 (0.5)***	37.8 (0.6)***
Systolic blood pressure (mmHg)	119.9 (2.3)	117.7 (2)	118.3 (2.5)	118.5 (3.1)	119.9 (2.7)
Diastolic blood pressure (mmHg)	77 (4)	76.6 (4.1)	76.8 (4)	75.7 (4)	76 (4.2)
Respiratory frequency (minutes)	21.3 (1.5)	20.8 (1.5)	21.8 (2.1)	21.9 (1.1)	21.7 (1.7)

All P values were calculed by Student´s test and indicate comparisons between treatment groups (* p < 0.05; ** p < 0.01; *** p < 0.001).

^&^ Women received another dose of 80 mg isosorbide dinitrate or 100 mcg misoprostol.

The tolerance to the combined isosorbide dinitrate-oxytocin regimen was adequate. Among the symptoms of nitrate overdose, headaches (likely because of a reduction in blood pressure) were recorded in six women (20%), with a non-significant risk of presenting with a headache (RR 1.5; 95% CI 0.5–4.8). For the misoprostol-oxytocin regimen, pelvic pain was recorded in 18 women (60%), with a high risk of pain (RR 8.6; 95% CI 2.3–35.4; p < 0.001) and an attributable risk of 53 per 100 women exposed to this therapeutic regimen. Only seven women (23.3%) presented with lumbar pain, and six of them (20%) presented with abdominal pain. No woman received treatment for likely or established sepsis nor was manual removal of the placenta necessary in any woman. No cases of tachysystole, hypertonicity, haemorrhage or coagulopathy were registered (Table 3).

**Table 3:** Non-serious adverse events reported by participants in the two treatment groups.

Adverse^&^	Combined regimen			
	Isosorbide dinitrate-oxytocin	Misoprostol-oxytocin	Relative Risk	P-value*
Headache	6 (20)	4 (13.3)	1.5 [0.5–4.8]	0.7
Abdominal pain	1 (3.3)	6 (20)	6 [0.8–46.9]	0.1
Pelvic pain	2 (6.6)	18 (60)	8.6 [2.3–35.4]	0.001
Backache	4 (13.3)	7 (23.3)	1.8 [0.6–5.4]	0.5

Values are given as n (%) or RR (95% CI).

^&^Nausea or vomiting not registrated.

*P-value by Fisher´s exact test.

Consent for a post-mortem examination of the foetus was obtained from only 10 women who participated in the study (16.7%). The determined causes of foetal death, according to the gynaecological classification for foetal deaths, were the following: unexplained in foetuses < 2,500 g, 30%; extrinsic foetal hypoxia, 10%; hydrops, 10%; and congenital anomalies, 50% (cardiac defects, defects of the duodenal tube, and total malformation). In women who had foetomaternal haemorrhaging, the problem was not suspected before the expulsion of the foetus. All patients received follow-up in hospital departments, and the durations of the hospital stays were significantly lower for the combination isosorbide dinitrate-oxytocin regimen than for the misoprostol-oxytocin regimen (31.9 ± 9.5 h vs. 39 ± 10.4 h, respectively; p < 0.008).

## Discussion

The availability of pharmacological agents to induce cervical ripening has contributed to the upward trend in the medical management of IUFD. Misoprostol is the most commonly used because of its relative stability at room temperature, easy administration and relatively low cost.

Although the administration of misoprostol alone or in combination with mifepristone shows high induction success rates [18], the use of prostaglandins and their analogues is limited by secondary effects related to the increase in the number of doses of misoprostol administered before effective uterine contractions are achieved. Side effects are the main disadvantage of misoprostol regimen and can occur in up to 30% of patients [19]. The most serious complications include abnormalities in uterine contractibility (tachysystole, hypertension and hyperstimulation), rapid separation of the placenta, post-partum haemorrhage and rare events, such as uterine rupture, amniotic liquid embolism, increased frequency of bowel movements, acidosis in new-borns and emergency caesarean sections because of foetal distress. Other adverse gastrointestinal effects include diarrhoea, abdominal pain, dyspepsia, flatulence, nausea and vomiting [20–23].

Based on sample size assumptions on log hazard ratio accuracy, we may infer with 95% confidence that the foetal expulsion rate using the isosorbide dinitrate-oxytocin combination is approximately 4.4 times, and at least 2.1 times, the foetal expulsion rate with the misoprostol-oxytocin regimen at any given point in time. The results of our study also demonstrate the efficacy of the combined isosorbide dinitrate-oxytocin regimen, with respective rates of successful uterine expulsions of 42%, 83.3% and 100% within 7, 10 and 15 h, respectively, and an average induction to delivery time of 8.7 h.

Other regimens with misoprostol have also been used successfully in the management of miscarriage, but no reports of isosorbide dinitrate-oxytocin use for IUFD cases are available, despite previous reports of the significant effect of isosorbide dinitrate in cervical ripening [9,24,25]. More than approximately half of all women undergoing labour and delivery after IUFD have an unfavourable cervix that requires some pharmacological agent for maturation, such as previous intracervical application of isosorbide dinitrate gel. Cervical ripening is characterised by decreased cervical collagen and an increase in hydration and active substances. The effects of isosorbide dinitrate may be attributable to direct or indirect actions on cervical cylindrical-epithelial cells [26]. Isosorbide dinitrate metabolites formed during denitrification—2-nitrate, 5-nitrate and isosorbide dinitrate—have short half-lives and act in the same manner as endothelium-derived relaxing factor (EDRF) on the molecular level. Increasing the cGMP concentration with EDRF has been accepted as a mediator of relaxation [27].

The most serious complication associated with the intravaginal use of NO donors for the induction of cervical ripening is headache because of increased maternal pulse. Nicoll et al. (2001) reported that pulse and maternal blood pressure were greater after the administration of isosorbide mononitrate (20 or 40 mg in tablets) compared to those in the group that was only examined vaginally [28]. In our study, the intravaginal administration of isosorbide dinitrate in gel solution followed by oxytocin had no significant adverse effect on patients’ headaches and/or haematological signs, including the loss of blood in the resolution of the IUFD pregnancy. However, 60% of the 30 women treated with the misoprostol-oxytocin regimen experienced more pelvic pain (RR 8.6; 95% CI 2.3‒35.4; p<0.001) with an attributable risk of 53 per every 100 women exposed to this therapeutic regimen. In previous studies in which isosorbide dinitrate gel solution was applied vaginally for induction of cervical ripening prior to first-trimester surgical evacuation of retained products of conception similar results have been observed [9]. No cases of uterine tachysystole, deep haemorrhaging, coagulopathy, nausea or vomiting were recorded.

The strengths of our study were the randomisation process used to ensure that the groups were comparable and that performance bias was minimised by blinding treatments and the use of clearly described treatment strategies. The adjustment of all baseline variables in the mathematical model not only avoided the possible confounding effect of the variable with respect to weeks of gestation at induction but also increased the power of the analysis of hazard functions. The Cox model was very useful for predicting the time-dependent outcome, and baseline variables showed no statistical correlation with the effects of the treatments. Therefore, the inclusion of the covariates maternal age, weeks of gestation and foetal weight and height allowed us to properly adjust the proportional hazards model and accurately estimate the foetal expulsion rates with reduced residual variance [29].

The main limitation of this study was the time required to recruit patients, which was attributed to the perception of the topic being sometimes considered ethically questionable. However, the women participants constituted a group that was willing to accept the treatment regime, which determined the outcomes. In conclusion, the medical management of labour after IUFD with the use of isosorbide dinitrate-oxytocin is an efficient, less invasive and safer strategy. In addition, intracervical application offers the advantage of the local administration of isosorbide dinitrate, which can contribute to decreasing the frequency and number of doses to and reduces adverse effects. This conclusion is the based on the evaluation of successful uterine expulsion rates and the average administration induction interval and comparison to published studies that utilised mifepristone-misoprostol, misoprostol-oxytocin or misoprostol alone.

## Supporting information

S1 Materials and Methods Compounds and substances that were purified or prepared especially for using in this study. (DOC)

S1 CONSORT checklist CONSORT 2010 checklist. (DOC)

S1 Protocol Study protocol approved by Institutional Review Board Ethical Committees. (DOC)
