# Supplementary material for: A randomized controlled trial comparing isosorbide dinitrate-oxytocin versus misoprostol-oxytocin at management of foetal intrauterine death
Source: PLoS One. 2019 Nov 21;14(11):e0215718. doi: 10.1371/journal.pone.0215718 (PMC6872136; doi:10.1371/journal.pone.0215718)
Supplement: S1 Materials and Methods — (DOC) [file pone.0215718.s001.doc]

**Supporting Materials and Methods S1**

**Elaboration of hydroxyethyl cellulose gel.** 2.5g hydroxyethyl cellulose (Janssen-Cilag, Puebla, México) were mixed in 100 mL hot water (50-60°C) with gently shaking until complete dispersion was achieved. Than, 5g propylene glycol were added and mixed to obtain a complete homogeneity at room temperature and it was transferred into a container until the following day. After said time, the gel was stirred to obtain a clear medium viscosity gel (1-2 minutes).

**Preparation of isosorbide dinitrate or misoprostol gel base.** A fine powder is prepared from porcelain mortar from Isosorbide dinitrate (Laboratorios Amstrong, Mexico City, Mexico) or misoprostol (Laboratorios Pfizer, Mexico City, Mexico). The fine powder reagent is transfered to a volumetric flask of appropriate volumen, and small portions of hydroxyethylcellulose hydrogel were added to obtain a homogeneous mixture with concentrations of 80 mg of isosorbide dinitrate or 100 µg of misoprostol in base gelled.

**Isosorbide Dinitrate and misoprostol evaluation test**

A base solution of isosorbide dinitrate and misoprostol was prepared to obtain a concentration of 20 μg/mL in 10% lactose solution. Serial dilutions were performed using known concentrations in mobile phase buffer solution (150 g ammonium acetate and 11.5 mL glacial acetic acid to 1 L water), methanol and water in a proportion of 350:100:550, respectively. The concentration of both drugs was determined using a UV spectrophotometer (Spectrophotometer UV, Beckman DU 65, CA, USA) reading at λ 220 nm and a reference filter of λ 278 nm. The mean absorbance for each set of standards, controls and samples was calculated by a standard plot curve. Computer-based curve-fitting statistical software was employed. Peak absorption and area under the curve were taken into account to determine stability of the pharmacological integration. Known samples of isosorbide dinitrate and misoprostol were added to 100% glycerin to prepare the gel solution. The reagent reservoirs had a final concentration of 80 mg isosorbide dinitrate and 100 µg misoprostol in 1.5 mL of gel solution.

References

Arteaga-Troncoso G, Villegas-Alvarado A, Belmont-Gomez A, Martinez-Herrera FJ, Villagrana-Zesati R, Guerra-Infante F. Intracervical application of the nitric oxide donor isosorbide dinitrate for induction of cervical ripening: a randomised controlled trial to determine clinical efficacy and safety prior to first trimester surgical evacuation of retained products of conception. BJOG 2005;112:1615‒1619.
