# Supplementary material for: A randomized controlled trial comparing isosorbide dinitrate-oxytocin versus misoprostol-oxytocin at management of foetal intrauterine death
Source: PLoS One. 2019 Nov 21;14(11):e0215718. doi: 10.1371/journal.pone.0215718 (PMC6872136; doi:10.1371/journal.pone.0215718)

**DATE:** November 27^th^, 2008

**NATIONAL INSTITUTE OF PERINATOLOGY**

**MONTES URALES #800**

**LOMAS VIRREYES**

**11000 MIGUEL HIDALGO**

**MEXICO CITY**

**MEXICO**

PROTOCOL REGISTRATION:

212250-29021

***PROTOCOL REGISTRATION***

**1. DATA.**

1.1. TITLE OF PROYECT.

**"Intravaginal application of isosorbide dinitrate gel solution to favor cervical ripening prior to induction of labour in women with late intrauterine death".**

1.2. RESEARCH DIVISION.

1.3. CLINICAL RESEARCH DIVISION.

1.4. RESEARCH PROGRAM IN MATERNAL MEDICINE.

1.5. START DATE: NOVEMBER, 2008

FINISH DATE: SEPTEMBER, 2011

1.6. MAIN RESEARCHER.

NAME: GABRIEL ARTEAGA TRONCOSO.

SIGNATURE _________________________________

ASCRIPTION: Department of Perinatal Infectology and Immunology

POSSITION: Medical Researcher

ACADEMIC LEVEL: Ph. D.

BELONGS TO NATIONAL INSTITUTE OF PERINATOLOGY: Yes

TO RESEARCH NATIONAL SYSTEM-CONACYT: Yes

***AUTHORIZATION DEPARTMENT.***

DEPARTMENT OF GYNAECOLOGY: Carlos Julián Neri Méndez, MD.

SIGNATURE_____________________________

MEDICAL SUBDIVISION: Edgar Hérnandez Andrade, Ph.D.

SIGNATURE _____________________________

RESEARCH DIVISION:

SIGNATURE _____________________________

1.7. CO-RESEARCHERS.

1.7.1.) NAME: CARLOS JULIAN NERI MENDEZ

ADSCRIPTION: Department of Gynaecology

POSSITION: Gynecologist

ACADEMIC LEVEL: Medical Doctor, specialist in obstetrics and gynaecology

BELONGS TO NATIONAL INSTITUTE OF PERINATOLOGY: Yes

TO RESEARCH NATIONAL SYSTEM-CONACYT: No

1.7.2.) NOMBRE: LOPÉZ HURTADO MARCELA

ADSCRIPTION: Department of Infectology and Immunology

POSSITION: Medical researcher

BELONGS TO NATIONAL INSTITUTE OF PERINATOLOGY: Yes

TO RESEARCH NATIONAL SYSTEM-CONACYT: Yes

1.7.3) NOMBRE: VILLAGRANA ZESATI JOSÉ ROBERTO

ADSCRIPTION: Department of Infectology and Immunology

POSSITION: Gynecologist

BELONGS TO NATIONAL INSTITUTE OF PERINATOLOGY: Yes

TO RESEARCH NATIONAL SYSTEM-CONACYT: No

1.7.4.) NOMBRE: ZEA PRADO FRANCISCO

ADSCRIPTION: Department of Gynaecology

POSSITION: Gynecologist

ACADEMIC LEVEL: Specialist in Gynecology and Obstetrics

BELONGS TO NATIONAL INSTITUTE OF PERINATOLOGY: Yes

TO RESEARCH NATIONAL SYSTEM-CONACYT: No

1.7.5.) NAME: BELMONT GÓMEZ AURORA

ADSCRIPTION: Clinical pharmacology

POSSITION: Medical pharmacologist

ACADEMIC LEVEL: Specialist in Gynecology and Obstetrics

BELONGS TO NATIONAL INSTITUTE OF PERINATOLOGY: Yes

TO RESEARCH NATIONAL SYSTEM-CONACYT: No

1.7.6.) NAME: GUERRA INFANTE FERNANDO MARTÍN

ADSCRIPTION: Department of Infectology and Immunology

POSSITION: Medical researcher

NIVEL ACADEMIC LEVEL: Ph D.

BELONGS TO NATIONAL INSTITUTE OF PERINATOLOGY: Yes

TO RESEARCH NATIONAL SYSTEM-CONACYT: Yes

- 1. PARTICIPATING DEPARTMENTS.

DEPARTMENT: Obstetrics and gynaecology

HEAD OF DEPARTMENT: Carlos Julián Neri Méndez, MD.

SIGNATURE _______________________________

DEPARTMENT: Clinical pharmacology

HEAD OF DEPARTMENT:. Aurora Belmont Gómez, MD.

SIGNATURE _______________________________

DEPARTMENT: Perinatal Infectology and Immunology

HEAD OF DEPARTMENT: Jesús Reyna Figueroa, MD.

SIGNATURE _______________________________

- 1. PARTICIPATING INSTITUTIONS.

INSTITUTION:

DATE CONVENTION:

1.10. TEACHING INTENTION

APPLICATIVE INTENTION:

TYPE OF THESIS: Basic studies ____ Specialty __X__ Subspecialty ______

Master in Science ______ Ph. D. Program____

SCHOOL:

AUTHORIZATION:

EDUCATION ADDRESS:

**2. THEORETICAL ASPECTS**

2.1. BACKGROUND

Foetal death is one of the saddest and most devastating episodes in gynaecology, in which the mother suffers an intense emotional crisis, along with the father and the family. A long delay in labour can increase the risk of anxiety and psychological anguish related to long-term symptoms. Women with little social support are particularly vulnerable to anxiety and postpartum depression.^1,2^

After the diagnosis of intrauterine foetal death (IUFD), the majority of hospitalised patients opt to deliver the baby within 48 hours.^3^ However, in women whose pregnancies are not gynaecologically monitored, spontaneous labour within three weeks of foetal death can result in complications for new pregnancies as a consequence of intrauterine infection. The induction of labour in a pregnant woman with an unripe cervical opening can be associated with the failed induction of labour and, invariably, with birth by caesarean section. The pharmacological induction of labour is an obstetric procedure that artificially initiates uterine concentrations that lead to progressive dilation and cervical ripening prior to the administration of intravenous oxytocin.^4^ For the induction of IUFD, methods of cervical preparation include the vaginal administration of dinoprostone, gemeprost, misoprostol, oxytocin, and progesterone receptor antagonists.^5,6^ The main problems cited by women during the induction of labour are excessive uterine activity and ineffective labour, which, in association with a history of placenta praevia or uterine surgery, can cause an increase in the risk of uterine rupture.^7,8^

The therapeutic efficacy and safety of nitric oxide donor drugs has been reported, with isosorbide dinitrate used to induce cervical ripening during the first trimester of gestation.^9^ Organic nitrates are reductively metabolised to release nitric oxide (NO), which stimulates the activation of soluble guanylyl cyclase (GC). This activation leads to the conversion of GTP into cyclic guanosine monophosphate (cGMP), the second messenger involved in NO mediation.^7^ The increase in cGMP levels causes the relaxation of the smooth musculature.^10-13^ In addition, the organic nitrates participate in the endogenous NO production pathway in endothelial cells to maintain vascular tone.^14-16^ This mechanism has led to an interest in exploring the metabolic activity of NO and medical use of nitrovasodilatory drugs as potential inducers of cervical ripening.

2.2. PROBLEM STATEMENT

The pre-induction of labor is an obstetrical procedure that is performed on women to provide a vaginal delivery in those with fetal death. The real benefit of the procedure is to avoid the risk of systemic infection in patients for performing a cesarean section. At present, the treatment indicated to start induction of labor has been established with the use of drugs like prostaglandins. Misoprostol and Dinoprostone have been widely administered during the last decade for inexpensive, easy to administer, effective and safe. However, Misoprostol, like other drug analogs is associated with adverse effects on the mother, especially abdominal pain, nausea, and vaginal bleeding leading to tachycardia that pregnancy is resolved through the abdomen emergency. It is also known that the frequency and intensity of these side effects are increased if the time interval in the pre-induction is prolonged.

At National Institute of Perinatology, the number of fetal deaths over 20 weeks of gestation has increased gradually. During the course of 1998 to 2006, mortality has increased from 95 cases to 211 in the period, with a fetal mortality rate of 38.6 per 1000 births for 2006. The procedures are often used to address labour in women with fetal death, to mention just a few examples, such as caesarean section or use of oxytocin without evidence of cervical ripening can contribute to increased maternal morbidity. In medical practice, using isosorbide dinitrate gel solution prior to induction of cervical dilation might reduce damage, economic cost of surgical procedures and risks to the health of the mother.

2.3. BIBLIOGRAPHIC HISTORY.

**2.3.1 The effect of nitric oxide donors on cervical ripening**

Endothelial cells produce and release vasoactive agents different regulating vascular smooth muscle tone. One such agent is a labile molecule called endothelium-derived relaxing factor (EDRF)^17^ and has been identified as nitric oxide (NO). The biochemical evidence shows that the vascular endothelium maintains some production of NO at baseline, but under special conditions NO acts as a potent endogenous vasodilator whose half life is very short. The paramagnetic properties of NO (odd number of electrons) contribute to having affinity for heme group which explains that is not associated with this group in the guanylate cyclase (GC).^18^ This binding causes a substantial increase in production Guanocín cyclic monophosphate (cGMP) in target endothelial cells occurs vascular.^19^ The vasorelaxation is produced from modulation of cGMP-dependent protein kinases, phosphorylation/dephosphorylation of myosin light chains and control of ion channels K^+^/Ca^+^ calcium homeostasis intracelular.^20,21^

Nitric oxide donors or organic compounds with ester functional group such as isosorbide dinitrate, glyceryl trinitrate (GTN) and pentaerythritol tetranitrate (PETN) have similar pharmacological properties, and like nitroglycerin are frequently prescribed in treating a variety of cardiac vascular disorders. Organic nitrates are reductively metabolised to release NO, which leads GC activation and catalysis in the conversion of GTP to the second messenger cGMP involved in mediating NO.^10^ Increased cGMP levels leads to relaxation of smooth muscle metabolism resulting NO.^11-13^ In addition, organic nitrates may participate in the endogenous pathway of NO production by endothelial cells to maintain tone vascular.^14-16^ This has led to interest in NO metabolic activity and clinical use of drugs as potential nitrovasodilator inducers of relaxation endocervical.^9^

**2.3.2 Cervical ripening**

The human cervix is ​​composed of a 10 to 15% of smooth muscle cells, and 85 to 90% of tissue conectivo.^22^ The vascular endothelium layer has a fine columnar epithelium that lines the channel containing a large amount endocervix branched glands and stroma shows an extracellular matrix, mainly collagen protein type I and III.^23,24^ addition, type IV collagen is present in smooth muscle cells and vessel walls sanguíneos.^25^ The collagen fibers provide stiffness that can be quickly replaced by collagenases, although the source and control of these enzymes have not yet been completely entendidas.^24^ The matrix is ​​composed of water, and protoglicosaminoglicanos glycosaminoglycans, as well as dermatan sulfate, heparan.^23^ hyaluronic acid and fibronectin Elastin sulfate and are located between the collagen fibers forming a thin layer beneath the epithelium proteins. Cervical ripening is the process that allows the cervix change of structure, designed to be tightly closed to maintain an intrauterine pregnancy, organ support expansion facilitating the passage of the fetus. A decrease in collagen content accompanied by an increase of water and hyaluronic acid allows softening of the uterine neck immediately before and during delivery. Structural changes showing the cervix during labor are defined in two phases: 1) maturation, showing the reorganization of collagen, and 2) expansion. Cervical ripening is the fundamental part of the stage that determines the birth, and occurs regardless of the uterinas.^23,26^ The internal bore of the cervix where cervical ripening process starts is shown in Figure 1. The cervical dilation involving a inflammatory reaction and the presence of a complex cascade of degradative enzymes accompanying rearrangement of extracellular matrix proteins and glicoproteínas.^23,27-29^ The hyperplasia and hypertrophy of fibroblasts, and tissue hydration growing in cervical region are anatomical and physiological changes that occur at the end of pregnancy.^23^

**2.3.3 Physiological aspects during cervical ripening**

Cervical ripening begins before the onset of contractions and labour. Cervical ripening is the result of a series of complex biochemical processes ending with the reorganization and restructuring of the collagen molecules where the cervix thins, softens, relaxes and dilates in response to the onset of uterine contractions. During last step of pregnancy, catabolic enzymes initiating the degradation of collagen and other structural proteins of outlayer matrix.^23,24^

The production of tumor necrosis factor (TNF-α) and interleukin 1 (IL-1β) induce increased the expression of endothelial adhesion molecules and extravasation of neutrophils into the cervical stroma body. It has also been found that high concentrations of hyaluronic acid correlate with induction of IL-1β and TNF-α.^30^ An increase in the concentration of IL-1β is associated with the expression of the messengers for interleukin-6 (IL-6) in cervix. IL-6 mRNA expression of interleukin 8 (IL-8) in the decidua of the corium and the presence of IL-1β and IL-8 in the amnion affect the beginning to labour.^31,32^ The presence of other cells like IL-8 that are located in the human cervix such as stromal origin, granulocyte macrophage can participate during labour.^31,33^ It should be noted that IL-8 levels of cervical origin correlate with collagenase release (matrix metalloprotease cervical-8) and this enzym which also modulate outlayer matrix proteins. ^31, 33-36^ Increased synthesis of IL-8 stimulates progesterone (PG) and leukotriene production causing dilation of blood vessels favoring the recruitment of polymorphonuclear cells.^30^ The presence of activated cells undergoing degranulation always accompanies lysis extracelullar.^23^ After matrix proteins degranulation by neutrophils proteases are a destabilized collagen fiber network. The action of enzymes on stromal cells is strictly limited on time and it is controlled by the concentration of inhibitory proteins present in tissue.^30^ Proteases matrix metalloproteinase 8 (MMP-8) seems to correlate with cervical ripening (localized mainly on stroma) unlike tissue matrix metalloproteinases 1 and 3.^29,37^ The use of exogenous interleukin 8 has been successful to ripen the cervix on some animal species.^38,39^

Moreover, the PG appears to be involved in controlling the maturation/cervical ripening especially the results obtained in animals and humans using anti-progestagens.^40,41^ Functional progesterone withdrawal conditions and prepares the start to delivery, ie, a change in the receptor-ligand affinity of PG and decreased concentration of the same in the myometrium and/or cervix. Preliminary results sugest the hypothesis that hormone withdrawal can take place in the human myometrium via changes in expression of co-activators progesterone receptor^42^ or the path of the differential expression of isoforms.^43,44^ These results support the idea that the disappearance of progesterone may occur through receptors, thereby facilitating the "feedback" of the hormone on pituitary gland. Other non-hormonal mediators involved in the maturation/cervical ripening include prostaglandins and IL-8^23,24^ and uterotoninas such as oxytocin and endothelin-1 that are independent mediators progesterone. Perhaps protease secretory leukocyte inhibitory (SLPI) that is present in the cervical mucus45 during maturation and which is a powerful inhibitor of neutrophil function with antagonistic action to the action of IL-8.^46^ In addition, activating factor platelets (PAF) is a pro-inflammatory cytokine which accelerates collagenolysis by pathway of induction of monocyte chemoattractant protein 1 (MCP-1) and on regulating RANTES T lymphocyte activation.^47^ Finally, several neuropeptides such as substance P, capsaicin, neurokinin A, gene-related peptide, calcitonin and secretoneurina belonging to the substances that can contribute efficiently on cervical ripening.^48^

**2.3.4 Nitric oxide is stimulated on cervix during labour**

The NO represents the end of the metabolic pathway in cervical ripening being accompanied by prostaglandin biosynthesis, and a high concentration of metabolic products of nitric oxide (NOx) have been identified in vaginal secretions during preterm labour.^49^ Although the source of these metabolites is unknown, it is possible that inflammatory cells infiltrating the as well tissue on the cervix, and this may be responsible for NO synthesis. The NO can induce cell death by apoptosis,^27,50^ but also activate MMPs.^51,52^ Thus, overproduction of NO can be related to cervical ripening, fragility of membranes and subsequent labour in premature birth.

Endogenous NO production was detected in the cervix of different species being negatively regulated during pregnancy and overregulated in labour. Studies in animals show that NO is associated with the induction of cervical ripening still high release of the molecule during labour.^53,54^ The NO share with TNF-α ability to initiate and/or block process of apoptosis depending on the cell cycle and its multiple factors that so far have not been identified.^50^ Although NO is an apoptotic substance that it can turn of the cellular cycle also permit the reorganization of collagen.^53,55^ Furthermore, NO in harmony with PGE2 can induce local vasodilation increasing vascular permeability and infiltration of leukocytes directly regulating the activity or production of MMPs.^56,57^Although, Ledingham et al. (1999) showed that the secretion of MMP-2 and MMP-9 in cervical fibroblasts were not covered by the overproduction of NO.^58^

***2.3.5 Endogenous synthesis of nitric oxide***

Endogenous NO is synthesized from the amino acid L-arginine and this synthesis is catalyzed by the enzyme nitric oxide synthase (NOS) in the presence of molecular oxygen, nicotinamide adenine dinucleotide phosphate (NADPH) and various cofactors tetrahidrobiopterinos. In addition, NO production is stimulated by intracellular calcium binding to calmodulin in endothelial cells. All three isoforms of nitric oxide synthase (nNOS, eNOS and iNOS) are present in the human cervix.^59-61^ The neuronal nitric oxide synthase (nNOS or NOS I) which is located in the stroma and epithelial cells,^61^ the inducible synthase (iNOS or NOS II) first identified in neurons, activated macrophages and vascular endothelial and epithelial cells and stroma of the spindle cervical cells, and endothelial enzyme (eNOS or NOS III) identified in vascular endothelium.^59^

Originally, NOS was discovered as an enzyme activator which catalyzes the synthesis of nitric oxide from L-arginine.^62^ Bredt et al. (1991) were the first researchers to publish the molecular cloning of NOS, indicating the existence of three binding sites for oxidative cofactors (NADPH, FAD, FMN) and the consensus sequence for a binding site calmodulin.^63^ The carboxy terminal sequence of NOS is like the cytochrome P450 reductase, and indeed, in the cerebellum NOS can function as a cytochrome P450 reductase.^64^ The cDNA of eNOS was the last of the three isoforms that was cloned to be highly homologous to other.^65^ The eNOS is a single copy of the genome located at chromosome encoded 7q35-36 in humans.^66^ The genomic DNA encoding a 1294 aa protein with a molecular weight of 135 kDa and it is expressed in endothelial cells that line the vasculature of connective tissue. The chemical activity of NO produced by the NOS pathway lies in the fact activate path soluble guanylate cyclase (sGC) causing increased synthesis of cGMP resulting intracellularly which in turn induces relaxation of subendothelial smooth muscle cells, including the veins in the brain and heart, and inhibits the adhesion and aggregation of platelets.^67^ Only, NOS type I and III are expressed constitutively calcium and calmodulin dependent, and requires the formation of iNOS from novo synthesis and calcium independently is mediated by certain cytokines such as IFN-γ and TNF-α, bacterial lipopolysaccharide processes and oxidative stress and inflammation.^68,69^

The transgenic mouse model of the eNOS allowed to explore the role of the enzyme in the regulation of blood pressure and different localization in tissues.^70-72^ These studies show overexpression sites of eNOS in vascular smooth muscle cells of the heart, lung and uterus, with lower levels of expression in brain, cerebellum and liver. However, the production of NO by NOS action in the endothelium of the animals is observed clearly reduced when mediated by nitrogenous vasodilators. These animals show a permanent state of hypotension, but without the increase in the volume of urine excreted or increased heart rate.

By contrast, "knock-out" mice for eNOS gene show hypertension phenotype with changes in behavior, heart valves level abnormalities, and insulin resistance; also they have high sensitivity to contractile action ß-adrenergic agonist. From these studies it has proposed the dual role of endothelial nitric oxide synthase as it is involved not only in regulating blood pressure, but also in lipid homeostasis, glucose and angiogenesis.^73-75^

***2.3.6 Nitric oxide donors and cervical ripening***

The GTN referred to as nitroglycerin was added as a therapeutic agent of angina pectoris. Other organic compounds with nitrate ester functional group (-O-NO2) as isosorbide dinitrate and pentaerythritol tetranitrate have similar pharmacological properties and like GTN are prescribed to treat a variety of heart and vascular damages.^76^

Organic nitrates are reduced to produce NO, which lead the activation of guanylate cyclase. The GC catalyzes the conversion of GTP to cGMP concentrations by increasing intracellular levels that lead to smooth muscle endothelial cell relaxation.^11-13,78-79^ Therefore, activity of the nitrate ester is related to the metabolism of NO and bioreactive these products can also participate in the endogenous pathway of NO production to maintain vascular smooth muscle tone.^14-16^ Previous studies indicate that bioactivation of nitrate ester to NO may be an enzymatic process which involve the cytochrome P-450 and/or thiol groups that require glutathione (GSH) to reduction.^80-84^ Moreover, nitrate ester activation may be mediated by the flavoprotein associated with the cell membrane.^85^ During chemical intimacy between thiol groups and flavins (flavin mononucleotide, FMN, flavin adenine dinucleotide, FAD) is possible the catalysis nitrate ester by NAD(P)H to reduce to different nitrogen species, which are procursores of NO formation.^86^

Chemical studies provide evidence that organic nitrate ester can be reduced in the presence of nucleotides and pirimídicos flavins nitrite ester. At the biochemical level, it is possible that this first step in the activation of nitrate ester is able to integrate the transmembrane flavoproteins (removal of radicals contributing to oxidative stress) a way that the nitrites potencialice catalyst for the production of NO. However, because nitrate ester more stable than nitrites to hydrolysis, the role of nitrate ester may be to act as a "shield" to the organic nitrite until it is fed completely to endothelial cell.^87,88^

After being formed, nitrite ester is hydrolyzed to give a hydrogen group a molecule with alcohol and inorganic nitrite, which reacts with GSH in the presence of glutathione S-transferase to form nitroglutationa S- (GSNO). This capability also integrates the idea that the glutathione S-transferase can be involved through its ability to catalyze the formation of S-nitrosothiols from GSH and endogenous nitrite ester. Based on these observations, a possible mechanism in the bioactivation of organic esters to induce relaxation of cervix is ​​summarized in Figure 2 and described under the following events: 1) The nitrates ester (RONO_2_) are taken up by smooth muscles endothelial cells; 2) These ester compounds diffuse within the cell being reduced to organic nitrates by action of some proteins bound to the membrane such as flavoproteins; 3) Reaction between GSH and nitrite ester (RONO) to form GSNO is catalyzed by GSH S-transferase, and; 4) The GSNO releases NO molecules by a variety of biochemical mechanisms not described yet on human cervix.^89,90^

Oxide nitric donors drugs are not effective to induce cervical ripening in animal models.45 Although NO donors such as isosorbide mononitrate,^56,91-95^ sodium nitroprusside,^96,97^ and glyceryl trinitrate applied intravaginally or via intracervical induce cervical ripening at the end of human pregnancy.^98,99^ In general, NO donors appear to be less effective to dilate the cervix that PGs in viable pregnancies. However, in nonviable pregnancies initial isosorbide dinitrate (IDN) has been more effective and safer than misoprostol for cervical ripening.^9^ The IDN and other oxide nitric donors are safe and show no major adverse effects on fetus and mothers.^28,91,100-104^

2.4. JUSTIFICATION

The pharmacological stimulation of cervical ripening in women with late intrauterine death attempts to reduce the number of caesarean sections performed in the medical units of gynecological care. The current pharmacological method for promoting induction of cervical ripening is by using prostaglandins that are generally administered in the posterior fornix of vagina. Evidence has shown histological changes on cervix induced by misoprostol and gemeprost that are similar in proline incorporation by sample protein and the diameter of the collagen fibers in certain tissue biopsies. The action of prostaglandins seems to be in the connective tissue stroma, and evidence of disintegration and dissolution of collagen are supported by the results of intake proline.^105^ Prostaglandins are not free of adverse effects, maternal and fetal events, mainly because of its stimulatory effect on uterine contractions. After application of prostaglandins for induction of cervical ripening, 5% of women observed uterine hypertonus. During labour, uterine hypertonia is not only painful for the mother, but can also result in fetal distress that often requires emergency caesarean section. Tachysystole and uterine hypertension associated with abnormal patterns of fetal heart rate have also been determine.^106^ In addition, these drugs have been associated with a number of side effects such as nausea, headache, vomiting, diarrhea, fever, chills, abdominal and vaginal bleeding.

Nitric oxide (NO)-releasing drugs are a novel class of effective and safe agents for cervical ripening such as isosorbide dinitrate. Several chemical donors of NO are currently being used in various types of experimental and therapeutic studies.^107^ The NO donor, isosorbide mononitrate, is effective at inducing cervical ripening during the first trimester, prior to medical termination of pregnancy.^108^ Other NO donors, such as glyceryl trinitrate and sodium nitroprusside, administered during the first trimester, have fewer adverse effects than the prostaglandin analogue gemeprost.^109^ In contrast to the stimulatory actions of prostaglandins, activation of the NO system leads to myometrial relaxation and can be used to successfully treat intrapartum fetal distress caused by uterine hypertonus.^110,111^ A fall in maternal blood pressure, if severe, will compromise uteroplacental blood flow causing fetal hypoxia. Nevertheless, the risk of maternal hypotension is a potential problem when using an NO-releasing drug for cervical ripening due to its vasodilatory function. Induction of cervical ripening preoperative reduces morbidity in surgical termination of pregnancy, including those related to hemorrhage, incomplete emptying of the uterus, uterine perforation and cervical trauma.

2.5. OBJECTIVE AND HYPOTHESIS

2.5.1. OBJECTIVE OF EXPERIMENT

The main objective of our study is to compare the efficacy and adverse effects of the intravaginal administration of isosorbide dinitrate in gel form followed by a high dose of oxytocin for the induction of labour after late foetal death with a commonly utilised combined regimen, misoprostol-oxytocin. Also, we will establish adverse events such as blood pressure, respiratory rate, and temperature, as well as nausea, headache, vomiting, diarrhea, fever, chills, abdominal pain and vaginal bleeding in women treated with misoprostol or isosorbide dinitrate.

2.5.2. HYPOTHESIS OF EXPERIMENT

It is expected that the 97% probability of achieving successful cervical ripening (Bishop score of over 7) with 80 mg of isosorbide dinitrate gel solution at 12 hours and a 67% chance of achieving successful cervical ripening with 100 mcg of misoprostol (power of 80%, α-value of 0.05, and β-value of 0.2) with 25% of all women whose will fail to induce labour. The main adverse effect associated with the administration of isosorbide dinitrate gel solution will be the headache and women treated with misoprostol will report preferably pelvic pain.

**3. DESIGN OF EXPERIMENTS**

3.1. PLANNING EXPERIMENT

EXPERIMENTAL STUDY _X_

CUASI-EXPERIMENTAL STUDY ___

OBSERVACIONAL STUDY ___

3.2 EXPERIMENTAL DESIGN

CLINICAL CONTROLLED TRIAL _X_

COHORT STUDY ___

CASE-CONTROL STUDY ___

CROSS-SECTIONAL STUDY ___

3.3. SOURCE OF VARIATION

RANDOMISED _X_ DESCRIPTIVE ___

LONGITUDINAL _X_ SECTIONAL ___

PROSPECTIVE _X_ RETROSPECTIVE ___

**4. METHODOLOGY**

4.1. PLACE AND TIME EXPERIMENT

The study will conducted in the obstetrics department of the “Isidro Espinosa de los Reyes” National Institute of Perinatology from November 2008 to September 2011. A prospective, randomised, double-blind, controlled clinical trial (RCT) will conducted to compare the efficacy and clinical safety of the induction of labour using the combination of isosorbide dinitrate-oxytocin (experimental arm, X=1) compared to misoprostol-oxytocin (standard arm, X=0). The drug´s characteristics that facilitates cervical ripening of women with intrauterine foetal death after 20 weeks of gestation and lack of uterine activity is clearly detailed in the following description and the accompanying tables and three figures highlight the site and mode of action of the drug as well as the recruitment and assignment of patients to each of the experimental groups.

4.2. UNIVERSE, EXPERIMENTAL UNITS, METHODS OF SAMPLING, AND SAMPLE SIZE.

4.2.1 UNIVERSE, AND EXPERIMENTAL UNITS

All pregnant women who come to National Institute of Perinatology.

All women with intrauterine foetal death after 20 weeks of gestation and lack of uterine activity, and to meet the criteria for inclusion.

4.3 DRUG DEVELOPMENT AND MANAGEMENT THERAPY

An isosorbide dinitrate- or misoprostol-based solution will prepared according to the previously published specifications.^9^ A base solution of isosorbide dinitrate and misoprostol will prepared to obtain a concentration of 20 mcg/mL in 10% lactose solution. Serial dilutions will performed using known concentrations in mobile phase buffer solution (150 g ammonium acetate and 11.5 mL glacial acetic acid to 1 L water), methanol and water in a proportion of 350:100:550, respectively. The concentration of both drugs will determined using a UV spectrophotometer (Spectrophotometer UV, Beckman DU 65, CA, USA) reading at E 220 nm and a reference filter of E 278 nm. The mean absorbance for each set of standards, controls and samples will calculated by a standard plot curve. Computer-based curve-fitting statistical software will employed. Peak absorption and area under the curve will taken into account to determine stability of the pharmacological integration. Known samples of isosorbide dinitrate and misoprostol will add to 100% glycerin to prepare the gel solution. The reagents will have final concentrations of either 80 mg of isosorbide dinitrate in 1.5 ml of gel solution or 100 mcg of misoprostol in the same presentation. Both solutions will packed in syringes that has the same appearance for the purpose of keeping the physician, patient and researcher blinded. The pharmacist will be the only participant who knew the contents of the syringes. Four syringes will placed in an opaque and sealed envelope consecutively numbered with a unique study number. The envelopes will open by the physician who applied the contents of the syringe, repeating the administration every 3 hours according to the prior selection and random allocation of patients. The entire "stock" of reagents remain stable for a period of 20 days and will maintain at room temperature (18-25° C) before using.

Opaque envelopes contained each microvial, and a consecutive number will assigned to each one and then sealed. These will randomly opened by the physician who applied the content of the microvial to the endocervix. Informed written consent will obtained and the study will be submitted for approval by the Ethics Committee of the National Institute of Perinatology.

4.3.1. ELIGIBILITY

Genders Eligible for Study: Female

Accepts Healthy Volunteers: No

CRITERIA

Inclusion Criteria:

- Closed cervix without evidence of cervical dilation or baseline uterine activity.
- A Bishop score of <5, having intact membranes.
- Gestation greater than or equal to 20 weeks established by the date of menstruation or by foetometry and ultrasound-confirmed late IUFD.

Exclusion Criteria:

- Multiple pregnancies.
- IUFD after late foeticide or the management of specific medical conditions associated with an increase in the risk of IUFD.
- Patients with a history of hypertension.
- Women with a history of unexplained antepartum haemorrhage, pelvic dystocia or any another counterindications where medications were used.

4.4. CHECKLIST (VARS)

**Patiens:**

Name

Age (years)

Gestational age (weeks)

Previous deliveries

Term deliveries

Preterm labour

Prior abortions

Uterine size (mm)

Long 1

Long 2

Transverse

**Haemodynamic stability:**

Heart rate (bpm)

Respiratory frequency (xminutes)

Core temperature (°C)

Systolic blood pressure (mmHg)

Diastolic blood pressure (mmHg)

**Adverse effects:**

Headache

Abdominal pain

Pelvic pain

Lower back pain

Nausea

Dizziness

Vomiting

**Main outcome measures:**

The main result of the study will the success rate of foetal expulsion within 15 hours, while the average administration induction interval defined the secondary result.

4.5. DATA COLLECTION

Data of the treatment will discarded, and only the study number will used to code the patient’s measurements. The type of treatment remained unknown to the participating women, and at the end of study period the results will analysed. All participants were given a vaginal exam by the same person, who was blinded to the treatment allocation. The medications were administered in the posterior fornix, and cervical activity was evaluated at baseline and every 3 hours to monitor any change based on the Bishop score.

If the cervical conditions did not change after the treatment application, participants will receive a new dose, without exceeding 4 doses, to facilitate cervical ripening. Once a Bishop score of over 7 will reached, oxytocin will infused in a balanced electrolyte solution beginning with an infusion rate of 2 mIU/min and doubling the dose every 15 minutes. The labour induction time from the first application of medication to the expulsion of the foetus will determined with a digital stopwatch. Each woman's vital signs will verified to determine that she is in stable condition and demonstrated haemodynamic stability as a requirement for the application of a new dose. During this time, the data and medical information will collected on paper and will later entered into a computational database. The participants will remain at rest during the evaluation of adverse effects, such as headache, abdominal pain, pelvic pain, lower back pain, nausea, dizziness, and vomiting. The lack of cervical activity after 4 doses of medication will considered treatment failure.

4.6. PILOT STUDY.

The purpose of conducting the pilot study will be examine the feasibility of an clinical approach study. Our pilot study can be used to evaluate the feasibility of recruitment, randomization, retention of patients and assessment procedures of the novel intervention. Ten women with ultrasonographic diagnosis of fetal death above 20 weeks gestation will be randomly selected to receive the pre-induction treatment based isosorbide dinitrate gel solution or misoprostol gel solution. The time interval between the application of pretreatment, the optimal relaxation of the cervix and conduction of labor will be determined in women in each experimental group. Also, clinical data obtained from these patients will serve to estimate the required sample size. Patients will have access to the best proven therapeutic method identiﬁed by the study.

4.7. STATISTICAL ANALYSIS.

Sample size calculations (Figure 3) will be based on a 97% probability of achieving successful than women undergoing pre-induction therapy with isosorbide dinitrate may reach a score> 7 on the scale of Bishop at 12 hours, and 67% chance for the group of women undergoing pre-treatment with misoprostol (α = 0.05, β = 0.2). Based on these assumptions, at least 30 women will be required for each treatment group. We believe this difference based on the results observed in a preliminary way. Values ​​are presented as mean ± SD. Both IDN and MIS will be evaluated to discern the clinical efficacy using the log rank test and Kaplan-Meier. Hemodynamic data obtained from each experimental group will be analyzed by the Student t test for independent samples. The drug safety will be evaluated based on the data of adverse effects by bivariate analysis (relative risks with 95% confidence interval). The differences in signs and symptoms between groups will be determined by chi-square. P <0.05 will be considered statistically significant. All statistical analyzes will be performed using SPSS for Windows (version 10.0, SPSS Inc., Chicago, USA).

4.8. ETHICAL ASPECTS

I. Research without risk ____

II. Research with low risk __X__

III. Research with high risk ____

| **TEXT PLEA**  **INFORMED CONSENT**  **I _________________________________________________________________**  **(Name of participants or their legal representative)**  freely declare that I agree to participate (in participating my client named below) in this research aim, procedures, benefits, and risks specified in this document.  It is my understanding that the researchers have provided me with any questions or answer any questions at the time of signing this, had not expressed or arising in the course of the investigation.  I have shown me that I can withdraw my consent to participate at any time without implying that the medical care that I provide, are affected by this.  I have been informed that participating in this study will not affect the cost of the drug that facilitates cervical relaxation, but if the total cost of the medical care I need to provide for labor and all information is given on my (his) identity and participation will remain confidential, except when authorized.  For the purposes it deems appropriate, I sign this by the researcher who reported me and two witnesses, maintaining a copy of a) Informed consent b) Information provided for my release. |
| --- |
|  |
| **DATE:** |
|  |
| NAME OF PARTICIPANT OR LEGAL REPRESENTATIVE |
|  |
| NAME OF RESEARCHER |
| FIRST WITNESS |
| SECOND WITNESS |

**5. ORGANIZATION.**

5.1. HUMAN RESOURCES AND MATERIALS

5.2. TRAINING OF PERSONNEL

5.2.1. TRAINING OF PERSONNEL:

5.2.2. TRAINING COURSES:

5.3. FINANCING

INTERNAL __________________ EXTERNAL ____________X____________

Budget format (next page)

In case of external financing, indicate source and amount

SOURCE: CONACYT grant 089486.

AMOUNT: $100 000.00

**B U D G E T**

| **PROTOCOL REGISTRATION: 212250-29021** |
| --- |

**Main researcher: Gabriel Arteaga Troncoso**

| **Sub-account** | **CONCEPT** | **NUMBER** | **UNIT COST (patient, case, sample or survey)** | **TOTAL** |
| --- | --- | --- | --- | --- |
| **01** | Research Unit |  |  |  |
|  | 01.1. patients |  |  |  |
|  | 01.1.1. Hospitalization day/bed |  |  |  |
|  | 01.1.2. medical consultation |  |  |  |
|  | 01.2. Other (specify) |  |  |  |
| **02** | Support Staff |  |  |  |
|  | 02.1. Doctor |  |  |  |
|  | 02.2. Nursing |  |  |  |
|  | 02.3. Secretarial assistant |  |  |  |
|  | 02.4. Data collector |  |  |  |
|  | 02.5. Interviewer |  |  |  |
|  | 02.6. Other (specify) |  |  |  |
| **03** | Laboratory exams |  |  |  |
|  |  |  |  |  |
|  |  |  |  |  |
| **04** | Desk studies |  |  |  |
|  |  |  |  |  |
|  |  |  |  |  |
| **05** | Special studies (describe) |  |  |  |
|  |  |  |  |  |
|  |  |  |  |  |
| **06** | Materials and equipment |  | Drugs and laboratory reagents,  publication of paper,  international congress and  national meeting | $100 000.00 |
|  |  |  |  |  |
|  |  |  |  |  |
| **07** | Researcher fees |  |  |  |
|  |  |  |  |  |
|  |  |  | **DIRECT COST** | $100 000.00 |
| **08** | National Institute of Perinatology (20% of direct costs) for externally funded projects | | |  |
|  |  |  |  |  |
|  |  |  | **TOTAL** | $100 000.00 |

**6. SCHEDULE OF ACTIVITIES**

PROTOCOL REGISTRATION: 212250-29021

TITLE OF PROYECT: Intravaginal application of isosorbide dinitrate gel solution to favor cervical ripening prior to induction of labour in women with late intrauterine death.

#### START DATE: NOVEMBER, 2008

#### FINISH DATE: SEPTEMBER, 2011

|  |  |  | |  | |  | |  | |  | |  | |  | |  | |  | |  | |  | |  | |  | |  | |  | |  | |  | |  | |  | |  |
| --- | --- | --- | --- | --- | --- | --- | --- | --- | --- | --- | --- | --- | --- | --- | --- | --- | --- | --- | --- | --- | --- | --- | --- | --- | --- | --- | --- | --- | --- | --- | --- | --- | --- | --- | --- | --- | --- | --- | --- | --- |
|  |  |  | |  | |  | |  | |  | |  | |  | |  | |  | |  | |  | |  | |  | |  | |  | |  | |  | |  | |  | |  |
| **Nº.** | **ACTIVITIES** | | MONTH SCHEDULE | | | | | | | | | | | | | | | | | | | | | | | | | | | | | | | | | | | | | |
|  |  | | **1** | | **2** | | **3** | | **4** | | **5** | | **6** | | **7** | | **8** | | **9** | | **10** | | **11** | | **12** | | **13** | | **14** | | **15** | | **16** | | **17** | | **18** | |  | |
| 1 | **CHECKLIST FOR PLANNING EXPERIMENTS AND PROCESS** | | x | | **x** | | **x** | | **x** | | **x** | | **x** | | **x** | | **x** | | **x** | | **x** | | **x** | |  | |  | |  | |  | |  | |  | |  | |  | |
| 2 | **PILOT STUDY** | | **x** | | **x** | |  | |  | |  | |  | |  | |  | |  | |  | |  | |  | |  | |  | |  | |  | |  | |  | |  | |
| 3 | **VALIDATION OF INSTRUMENTS** | | **x** | | **x** | |  | |  | |  | |  | |  | |  | |  | |  | |  | |  | |  | |  | |  | |  | |  | |  | |  | |
| 4 | **DATA COLLECTION** | |  | |  | | **x** | | **x** | | **x** | | **x** | | **x** | | **x** | | **x** | | **x** | | **x** | | **x** | | **x** | | **x** | |  | |  | |  | |  | |  | |
| 5 | **CODING OF VARS** | |  | |  | | **x** | | **x** | | **x** | | **x** | | **x** | | **x** | | **x** | | **x** | | **x** | | **x** | | **x** | | **x** | |  | |  | |  | |  | |  | |
| 6 | **DATA PROCESSING** | |  | |  | |  | |  | |  | |  | |  | |  | |  | |  | |  | |  | |  | | **x** | | **x** | |  | |  | |  | |  | |
| 7 | **ANALYSIS OF DATA** | |  | |  | |  | |  | |  | |  | |  | |  | |  | |  | |  | |  | |  | | **x** | | **x** | |  | |  | |  | |  | |
| 8 | **DRAFTING OF THE FINAL REPORT** | |  | |  | |  | |  | |  | |  | |  | |  | |  | |  | |  | |  | |  | |  | | **x** | | **x** | | **x** | | **x** | |  | |
| 9 | **DEVELOPMENT OF MANUSCRIPT** | |  | |  | |  | |  | |  | |  | |  | |  | |  | |  | |  | |  | |  | |  | | **x** | | **x** | | **x** | | **x** | |  | |

**REFERENCES**

1. Koopmans L, Wilson T, Cacciatore J, Flenady V. Support for mothers, fathers and families after perinatal death. Cochrane Database Syst Rev 2013;6:CD000452. doi: 10.1002/14651858.CD000452.pub3.
2. Rådestad I, Steineck G, Nordin C, Sjögren B. Psychological complications after stillbirth--influence of memories and immediate management: population based study. BMJ 1996;312:1505-8.
3. Silver RM, Heuser CC. Stillbirth workup and delivery management. Clin Obstet Gynecol 2010;53:681-90.
4. World Health Organization. Induction and augmentation of labour. In: WHO, UNFPA, UNICEF, World Bank, editor. Managing Complications in Pregnancy and Childbirth: A Guide for Midwives and Doctors. Geneva: WHO; 2000. pp. 17-25.
5. Hughes EG, Kelly AJ, Kavanagh J. Dinoprostone vaginal insert for cervical ripening and labor induction: a meta-analysis. Obstet Gynecol 2001;97:847-55.
6. Neiger R, Greaves PC. Comparison between vaginal misoprostol and cervical dinoprostone for cervical ripening and labor induction. Tenn Med 2001;94:25-7.
7. Bolnick JM, Velazquez MD, Gonzalez JL, Rappaport VJ, McIlwain-Dunivan G, Rayburn WF. Randomized trial between two active labor management protocols in the presence of an unfavorable cervix. Am J Obstet Gynecol 2004;190:124-8.
8. Khan RU, El-Refaey H, Sharma S, Sooranna D, Stafford M. Oral, rectal, and vaginal pharmacokinetics of misoprostol. Obstet Gynecol 2004;103:866-70.
9. Arteaga-Troncoso G, Villegas-Alvarado A, Belmont-Gomez A, Martinez-Herrera FJ, Villagrana-Zesati R, Guerra-Infante F. Intracervical application of the nitric oxide donor isosorbide dinitrate for induction of cervical ripening: a randomised controlled trial to determine clinical efficacy and safety prior to first trimester surgical evacuation of retained products of conception. BJOG 2005;112:1615-9.
10. Ignarro LJ, Lippton H, Edwards JC, Baricos WH, Hyman AL, Kadowitz PJ, et al. Mechanism of vascular smooth muscle relaxation by organic nitrates, nitrites, nitroprusside and nitric oxide: evidence for the involvement of S-nitrosothiols as active intermediates. J Pharmacol Exp Ther 1981;218:739-49.
11. Ignarro LJ. Heme-dependent activation of soluble guanylate cyclase by nitric oxide: regulation of enzyme activity by porphyrins and metalloporphyrins. Semin Hematol 1989;26:63-76.
12. Schmidt HH, Lohmann SM, Walter U. The nitric oxide and cGMP signal transduction system: regulation and mechanism of action. Biochim Biophys Acta 1993;1178:153-75.
13. Murad F. Regulation of cytosolic guanylyl cyclase by nitric oxide: the NO-cyclic GMP signal transduction system. Adv Pharmacol 1994;26:19-33.
14. Chung SJ, Fung HL. Identification of the subcellular site for nitroglycerin metabolism to nitric oxide in bovine coronary smooth muscle cells. J Pharmacol Exp Ther 1990;253:614-9.
15. Feelisch M, Kelm M. Biotransformation of organic nitrates to nitric oxide by vascular smooth muscle and endothelial cells. Biochem Biophys Res Commun 1991;180:286-93.
16. Michel T, Smith TW. Nitric oxide synthases and cardiovascular signaling. Am J Cardiol 1993;72:33C-8.
17. Furchgott RF and Zawadzki JV. The obligatory role of endotelial cells in the relaxation of arterial smooth muscle by acethylcholine. Nature 1980; 288: 373-376.
18. Ignarro LJ. Biosynthesis and metabolism of endothelium-derived nitric oxide. Annu Rev Pharmacol Toxicol 1990; 30: 535-560.
19. Craven PA and De Rubertis FR. Restoration of the responsiveness of purified guanylate cyclase to nitrosoguanidine, nitric oxide and related activators by heme and hemeproteins: Evidence for involvement of the paramagnetic nitrosyl heme complex in enzyme activation. J Biol Chem 1978; 253: 8433-8443.
20. Rapaport RM, Draznin MB and Murad F. Endothelium-dependent vasodilator- and nitrovasodilator-induced relaxation may be mediated through cyclic GMP formation and cyclic GMP-dependent protein phosphorylation. Trans Ass Am Physicians 1983; 96: 19-30.
21. Moncada S and Higgs EA. Endogenous nitric oxide physiology, pathology and clinical relevance. Eur J Clin Invest 1991; 21: 361-374.
22. Danforth DN. The morphology of the human cervix. *Clin Obstet Gynecol* 1983; 26**:** 7-13.
23. Leppert PC. Anatomy and physiology of cervical ripening. *Clin Obstet Gynecol* 1995; 38**:** 267-279.
24. Kelly RW. Inflammatory mediators and cervical ripening. *J Reprod Immunol* 2002; 57**:** 217-224.
25. Minamoto T, Arai K, Hirakawa S and Nagai Y. Immunohistochemical studies on collagen types in the uterine cervix in pregnant and nonpregnant states. *Am J Obstet Gynecol* 1987; 156**:** 138-144.
26. Chwalisz K and Garfield RE. Role of nitric oxide in the uterus and cervix: implications for the management of labor. *J Perinat Med* 1998; 26**:** 448-457.
27. Leppert PC. Proliferation and apoptosis of fibroblasts and smooth muscle cells in rat uterine cervix throughout gestation and the effect of the antiprogesterone onapristone. *Am J Obstet Gynecol* 1998; 178**:** 713-725.
28. Maul H, Longo M, Saade GR and Garfield RE. Nitric oxide and its role during pregnancy: from ovulation to delivery. *Curr Pharm Des* 2003; 9**:** 359-380.
29. Sennström MB, Brauner A, Byström B, Malmström A and Ekman G. Matrix metalloproteinase-8 correlates with the cervical ripening process in humans. *Acta Obstet Gynecol Scand* 2003; 82**:** 904-911.
30. Winkler M and Rath W. Changes in the cervical extracellular matrix during pregnancy and parturition. *J Perinat Med* 1999; 27**:** 45-60.
31. Osman I, Young A, Ledingham MA, Thomson AJ, Jordan F, Greer IA and Norman JE. Leukocyte density and pro-inflammatory cytokine expression in human fetal membranes, decidua, cervix and myometrium before and during labour at term. *Mol Hum Reprod* 2003; 9**:** 41-45.
32. Sennström MB, Ekman G, Westergren-Thorsson G, Malmström A, Byström B, Endresen U, et al. Human cervical ripening, an inflammatory process mediated by cytokines. *Mol Hum Reprod* 2000; 6**:** 375-381.
33. Sakamoto Y, Moran P, Searle RF, Bulmer JN and Robson SC. Interleukin-8 is involved in cervical dilatation but not in prelabour cervical ripening. *Clin Exp Immunol* 2004; 138**:** 151-157.
34. Osmers, R.G., Adelmann-Grill, B.C., Rath, W., Stuhlsatz, H.W., Tschesche, H. and Kuhn, W. 1995a. Biochemical events in cervical ripening dilatation during pregnancy and parturition. *J Obstet Gynecol* 1995; 21**:** 185-194.
35. Osmers RG, Blaser J, Kuhn W and Tschesche H. Interleukin-8 synthesis and the onset of labor. *Obstet Gynecol* 1995; 86**:** 223-229.
36. Sennström MB, Brauner A, Lu Y, Granström LM, Malmström AL and Ekman GE. Interleukin-8 is a mediator of the final cervical ripening in humans. *Eur J Obstet Gynecol Reprod Biol* 1997; 74**:** 89-92.
37. Aronsson A, Ulfgren AK Stabi B, Stavreus-Evers A and Gemzell-Danielsson K. The effect of orally and vaginally administered misoprostol on inflammatory mediators and cervical ripening during early pregnancy. *Contraception* 2005; 72**:** 33-39.
38. Garcia-Velasco JA and Arici A. Chemokines and human reproduction. *Fertil Steril* 1999; 71**:** 983-993.
39. Chwalisz K, Benson M, Scholz P, Daum J, Beier HM and Hegele-Hartung C. Cervical ripening with the cytokines interleukin 8, interleukin 1 beta and tumour necrosis factor alpha in guinea-pigs. *Hum Reprod* 1994; 9**:** 2173-2181.
40. Garfield RE, Saade G, Buhimschi C, Buhimschi I, Shi L, Shi SQ and Chwalisz, K. Control and assessment of the uterus and cervix during pregnancy and labour. *Hum Reprod Update* 1998; 4**:** 673-695.
41. Neilson JP. Mifepristone for induction of labour. Cochrane Database of Systematic Reviews 2004, 4.
42. Condon JC, Jeyasuria P, Faust JM, Wilson JW and Mendelson CR. A decline in the levels of progesterone receptor coactivators in the pregnant uterus at term may antagonize progesterone receptor function and contribute to the initiation of parturition. *Proc Natl Acad Sci USA* 2003; 100**:** 9518-9523.
43. Madsen, G., Zakar, T., Ku, C.Y., Sanborn, B.M., Smith, R. and Mesiano, S. Prostaglandins differentially modulate progesterone receptor-A and -B expression in human myometrial cells: evidence for prostaglandin-induced functional progesterone withdrawal. *J Clin Endocrinol Metab* 2004; 89**:** 1010-1013.
44. Stjernholm-Vladic Y, Stygar D, Månsson C, Masironi B, Åkerberg S, Wang H, Ekman-Ördeberg G and Sahlin L. Factors involved in the inflammatory events of cervical ripening in humans. *Reprod Biol Endocrinol* 2004; 2**:** 74.
45. Denison FC, Calder AA and Kelly RW. The action of prostaglandin E2 on the human cervix: stimulation of interleukin 8 and inhibition of secretory leukocyte protease inhibitor. *Am J Obstet Gynecol* 1999; 180: 614-620.
46. Sallenave JM, Si Tahar M, Cox G, Chignard M and Gauldie J. Secretory leukocyte proteinase inhibitor is a major leukocyte elastase inhibitor in human neutrophils. *J Leukoc Biol* 1997; 61: 695-702.
47. Sugano T, Narahara H, Nasu K, Arima K, Fujisawa K and Miyakawa I. Effects of plateletactivating factor on cytokine production by human uterine cervical fibroblasts. *Mol Hum Reprod* 2001; 7**:** 475-481.
48. Collins JJ, Usip S, McCarson KE and Papka RE. Sensory nerves and neuropeptides in uterine cervical ripening. *Peptides* 2002; 23**:** 167-183.
49. Nakatsuka M, Habara T, Kamada Y, Tada K and Kudo T. Elevation of total nitrite and nitrate concentration in vaginal secretions as a predictor of premature delivery. *Am J Obstet Gynecol* 2000; 182: 644-645.
50. Brune B, von Knethen A and Sandau KB. Nitric oxide and its role in apoptosis. *Eur J Pharmacol* 1998; 351: 261-272.
51. Yoshida M, Sagawa N, Itoh H, Yura S, Korita D, Kakui K, Hirota N, Sato T, Ito A and Fujii S. Nitric oxide increases matrix metalloproteinase-1 production in human uterine cervical fibroblast cells. *Mol Hum Reprod* 2001; 7**:** 979-985.
52. Biondi C, Pavan B, Lunghi L, Fiorini S and Vesce F. The role and modulation of the oxidative balance in pregnancy. *Curr Pharm Des* 2005; 11**:** 2075-2089.
53. Chwalisz K and Garfield RE. Regulation of the uterus and cervix during pregnancy and labor. Role of progesterone and nitric oxide. *Ann NY Acad Sci* 1997; 828**:** 238-253.
54. Buhimschi I, Ali M, Jain V, Chwalisz K and Garfield RE. Differential regulation of nitric oxide in the rat uterus and cervix during pregnancy and labour. *Hum Reprod* 1996; 11**:** 1755-1766.
55. Leppert PC, Kokenyesi R, Klemenich CA and Fisher J. Further evidence of a decorin-collagen interaction in the disruption of cervical collagen fibers during rat gestation. *Am J Obstet Gynecol*  2000; 182**:** 805-811.
56. Ekerhövd E, Weijdegard B, Brännström M, Mattsby-Baltzer I and Norström A. Nitric oxide induced cervical ripening in the human: Involvement of cyclic guanosine monophosphate, prostaglandin F(2 alpha), and prostaglandin E(2). *Am J Obstet Gynecol*  2002; 186**:** 745-750.
57. Maul H, Longo M, Saade GR and Garfield RE. The physiology of uterine contractions. Clin Perinatol 2003; 30: 665-676
58. Ledingham MA, Denison FC, Riley SC and Norman JE. Matrix metalloproteinases-2 and -9 and their inhibitors are produced by the human uterine cervix but their secretion is not regulated by nitric oxide donors. *Hum Reprod* 1999; 14**:** 2089-2096.
59. Tschugguel W, Schneeberger C, Lass H, Stonek F, Zaghlula MB, Czerwenka K, et al. Human cervical ripening is associated with an increase in cervical inducible nitric oxide synthase expression. *Biol Reprod* 1999; 60**:** 1367-1372.
60. Ledingham MA, Thomson AJ, Young A, Macara, LM, Greer IA and Norman JE. Changes in the expression of nitric oxide synthase in the human uterine cervix during pregnancy and parturition. *Mol Hum Reprod* 2000; 6**:** 1041-1048.
61. Bao S, Rai J and Schreiber J. Brain nitric oxide synthase expression is enhanced in the human cervix in labor. *J Soc Gynecol Investig* 2001; 8**:** 158-164.
62. Palmer RMJ, Ashton DS and Moncada S. Vascular endothelial cells synthesize nitric oxide from L-arginine. Nature 1988; 333: 664-666.
63. Bredt DS, Hwang PM, Glatt CE, Lowenstein C, Reed RR y Snyder SH: Cloned and expresed nitric oxide synthase structurally resembles cytochrome P-450 reductase. Nature 1991; 351: 714-718.
64. Klatt P, Heinzel B, John M, Kastner M, Bohme E y Mayer B. Ca2+/calmodulin-dependent cytochrome C reductase activity of brain nitric oxide synthase. J Biol Chem 1992; 267: 11374-11378.
65. Nathan C and Xie QW. Nitric oxide synthases: roles, tolls, and controls. Cell 1994; 78: 915-918.
66. Marsden PA, Heng HHQ, Scherer SW. Structure and chromosomal localization of the human constitutive endothelial NO synthase. J Biol Chem 1993; 268: 17478-17488.
67. Kenneth KW. Regulation of endothelial nitric oxide synthase activity and gene expression. In: Nitric Oxide. Novel actions, deleterious effects, and clinical potential. Eds. Chiueh, C.C., Hong, J-S., Kee, L. S. Annals New York Acad Sc 962: 122-130, 2002.
68. Ding Y, Vaziri ND. Calcium channel blockade enhances nitric oxide synthase expression by cultured endothelial cells. Hypertension 1998; 32: 718–723.
69. Rodríguez-Mañas L, Sánchez-Rodríguez C, Vallejo S, El-Assar M, Peiró C, Azcutia V, Matesanz N, Sánchez-Ferrer CF and Nevado J. Pro-inflammatory effects of early non-enzymatic glycated proteins in human mesothelial cells vary with cell donor’s age. British J Pharmacology 2006; 149, 979-987.
70. Ohashi Y, kawashima S, Hirata K, Yamashita T, Ishida T, Inoue N, et al. Hypotension and reduced nitric oxide-elicted vasorelaxation in transgenic mice over expressing endothelial nitric oxide synthase. J Clin Invest 1998; 102: 2061-2071.
71. Bader M. Transgenic animal models for the functional analysis of vasoactive peptides. Braz J Med Biol Res 1998; 31: 1171-1183.
72. Haperen RV, Cheng C, Mess BM, Van deel E, Waard M, Van Damme LCA, et al. Functional expression of endothelial nitric oxide synthase fused to green fluorescent protein in transgenic mice. Am J Pathol 2003; 163: 1677-1686.
73. Huang PL,Huang Z, Mashimo H, Bloch KD, Moskowitz MA, Bevan JA, et al. Hypertension in mice lacking the gene for endothelial nitric oxide synthase. Nature 1995; 377: 239-242.
74. Shesely EG, Maeda N, Kim HS, Desai KM, Krege JH, Laubach VE, et al. Elevated blood pressures in mice lacking endothelial nitric oxide synthase. Proc Natl Acad Sci USA 1996; 93:13176-13181.
75. Albertech E, Stegeman CA, Herringa P, Henning RH, Van Goor H. Protective role of endothelial nitric oxide synthase. J Pathol 2003; 199: 8-17.
76. Katzung BG and Chatterjee K. Vasodilation and the treatment of angina pectoris. In Basic and Clinical Pharmacology (Katzung BG ed.) p 1017, Appleton and Lange, Norwalk, CT., 1989.
77. Arnold WP, Mittal CK, Katsuki, S and Murad F. Nitric oxide activates guanylate ciclase and increases 3´,5´-monophosphate levels in various tissue preparations. Proc Natl Acad Sci USA 1977; 74: 3203- 3207.
78. Ignarro LJ, Barry BK, Gruetter DY, Ohlstein EH, Gruetter CA, Kadowitz PJ and Baricos WH. Selective alterations in responsiveness of guanylate cyclase to activation by nitroso compounds during enzyme purification. Biochim Biophys Acta 1981; 673: 394-407.
79. Kurz MA, Lamping KG, Bates JN, Eastham CL, Marcus ML and Harrison DG. Mechanisms responsible for the heterogeneous coronary microvascular response to nitroglycerin. Circ Res 1991; 68: 847-855.
80. Servent D, Delaforge M, Ducrocq C, Mansuy D and Lenfant M. Nitric oxide formation during microsomal hepatic denitration of glyceryl trinitrate: Involvement of cytochrome P-450. Biochem Biophys Res Commun 1989; 163:1210-1216.
81. Simon WC, Anderson DJ and Bennett BM. Inhibition of the pharmacological actions of glyceryl trinitrate after the electroporetic delivery of a glutathione S-transferase inhibitor. J Pharmacol Exp Ther 1996; 279:1535-1540.
82. Schroder H. Cytochrome P-450 mediates bioactivation of organic nitrates. J Pharmacol Exp Ther 1992; 262:298-302.
83. Nigam R, Whiting T and Bennett BM. Effect of inhibitors of glutathione S-transferase on glyceryl trinitrate activity in isolated rat aorta. Can J Physiol Pharmacol 1993; 71:179-184.
84. Nigam R, Anderson DJ, Lee SF and Bennett BM. Isoform-specific biotransformation of glyceryl trinitrate by rat aortic glutathione S-transferases. J Pharmacol Exp Ther 1996; 279:1527-1534.
85. McGuire JJ, Anderson DJ and Bennett BM (1994) Inhibition of the biotransformation and pharmacological actions of glyceryl trinitrate by the flavoprotein inhibitor, diphenyleneiodonium sulfate. J Pharmacol Exp Ther 1994; 271: 708-714.
86. Massey V. The chemical and biological versatility of riboflavin. Biochem Soc Trans 2000; 28: 283-296.
87. Allen AD. Hydrolysis and alcoholysis of some organic nitrites. J Chem Soc 1952 (London), 1193-1206.
88. Baker JW and Easty DM (1952) Hydrolytic decomposition of esters of nitric acid. J Chem Soc 1952 (London), 1968-1974.
89. Seth P and Fung HL. Biochemical characterization of a membrane-bound enzyme responsible for generating nitric oxide from nitroglycerin in vascular smooth muscle cells. Biochem Pharmacol 1993; 46: 1481-1486.
90. Wong S-YP and Fukuto MJ. Reaction of organic nitrate esters and s-nitrosothiols with reduced flavins: A possible mechanism of bioactivation. Drug Metabol D 1999; 27: 502-509.
91. Thomson, A., Lunan, C., Ledingham, M., Howat, R., Cameron, I., Greer, I. and Norman, J. 1998. Randomised trial of nitric oxide donor versus prostaglandin for cervical ripening before first-trimester termination of pregnancy. Lancet 352: 1093-1096.
92. Nicoll AE, Mackenzie F, Greer IA and Norman JE. Vaginal application of the nitric oxide donor isosorbide mononitrate for preinduction cervical ripening: a randomized controlled trial to determine effects on maternal and fetal hemodynamics. Am J Obstet Gynecol 2001; 184: 958-964.
93. Li C, Chan C and Ho P. A comparison of isosorbide mononitrate and misoprostol cervical ripening before suction evacuation. Obstet Gynecol 2003; 102: 583-588.
94. Li C, Chan C and Ho P. A study of the efficacy of cervical ripening with nitric oxide donor versus placebo for cervical priming before second-trimester termination of pregnancy. Contraception 2003; 68: 269-272.
95. Eppel W, Facchinetti F, Schleussner E, Piccinini F, Pizzi C, Gruber DM, et al. Second trimester abortion using isosorbide mononitrate in addition to gemeprost compared with gemeprost alone: a double-blind randomized, placebo-controlled multicenter trial. Am J Obstet Gynecol 2005; 192: 856-861.
96. Facchinetti F, Piccinini F and Volpe A. Chemical ripening of the cervix with intracervical application of sodium nitroprusside: a randomized controlled trial. Hum Reprod 2000; 15: 2224-2227.
97. Chan CC, Tang OS, Ng EH, Li CF and Ho PC. Intracervical sodium nitroprusside versus vaginal misoprostol in first trimester surgical termination of pregnancy: a randomized double-blinded controlled trial. Hum Reprod 2005; 20: 829-833.
98. Chanrachakul B, Herabutya Y and Punyavachira P. Randomized comparison of glyceryl trinitrate and prostaglandin E2 for cervical ripening at term. Obstet Gynecol 2000; 96: 549-553.
99. Sharma Y, Kumar S, Mittal S, Misra R and Dadhwal V. Evaluation of glyceryl trinitrate, misoprostol, and prostaglandin E gel for preinduction cervical ripening in term pregnancy. J Obstet Gynaecol Res 2005; 31: 210-215.
100. Cacciatore B, Halmesmäki E, Kaaja R, Teramo K and Ylikorkala O. Effects of transdermal nitroglycerin on impedance to flow in the uterine, umbilical, and fetal middle cerebral arteries in pregnancies complicated by preeclampsia and intrauterine growth retardation. Am J Obstet Gynecol 1998; 179: 140-145.
101. Bates CD, Nicoll AE, Mullen AB, Mackenzie F, Thomson AJ and Norman JE. Serum profile of isosorbide mononitrate after vaginal administration in the third trimester. BJOG 2003; 110: 64-67.
102. Ekerhövd E, Bullarbo M, Andersch B and Norström A. Vaginal administration of the nitric oxide donor isosorbide mononitrate for cervical ripening at term: a randomized controlled study. Am J Obstet Gynecol 2003; 189: 1692-1697.
103. Kahler C, Schleussner E, Moller A and Seewald HJ. Nitric oxide donors: effects on fetoplacental blood flow. Eur J Obstet Gynecol Reprod Biol 2004; 115: 10-14.
104. De Pace V, Chiossi G and Facchinetti F. Clinical use of oxide donors and L-arginine in obstetrics. J Maternal-Fetal Neonatal Med 2007; 20: 569-579.
105. El-Refaey H, Calder L, Wheatley DN, Templeton A. Cervical ripening with prostaglandin E1 analogues, misoprostol and gemeprost. Lancet 1994; 343: 1207-1209.
106. Wing DA, Rahal A, Jones MM, Goodwin M, Paul RH. Misoprostol: an effective agent for cervical ripening and labor induction. Am J Obstet Gynecol 1997; 172: 1811-1816.
107. Änggård E. Nitricoxide:mediator,murderer,andmedicine. Lancet 1994;343:1199–1206.
108. Thomson AJ, Lunan CB, Cameron AD, et al. Nitric oxide donors induce ripening of the human uterine cervix: a randomised controlled trial. Br J Obstet Gynaecol 1997;104:1054–1057.
109. Thomson AJ, Lunan CB, Ledingham M, et al. Randomised trial of nitric oxide donor versus prostaglandin for cervical ripening before first trimester termination of pregnancy. Lancet 1998;352:1093–1096.
110. Mercier F, Dounas M, Bouaziz H, et al. Intravenous nitroglycerin to relive intrapartum fetal distress related to uterine hyperactivity: a prospective observational study. Anesth Analg 1997;84:1117–1120.
111. Norman, J.E., Ward, L.M., Martin, W., Cameron, A.D., McGrath, J.C., Greer, I.A. and Cameron, I.T. Effects of cGMP and the nitric oxide donors glyceryl trinitrate and sodium nitroprusside on contractions in vitro of isolated myometrial tissue from pregnant women. J Reprod Fertil 1997; 110: 249-254.


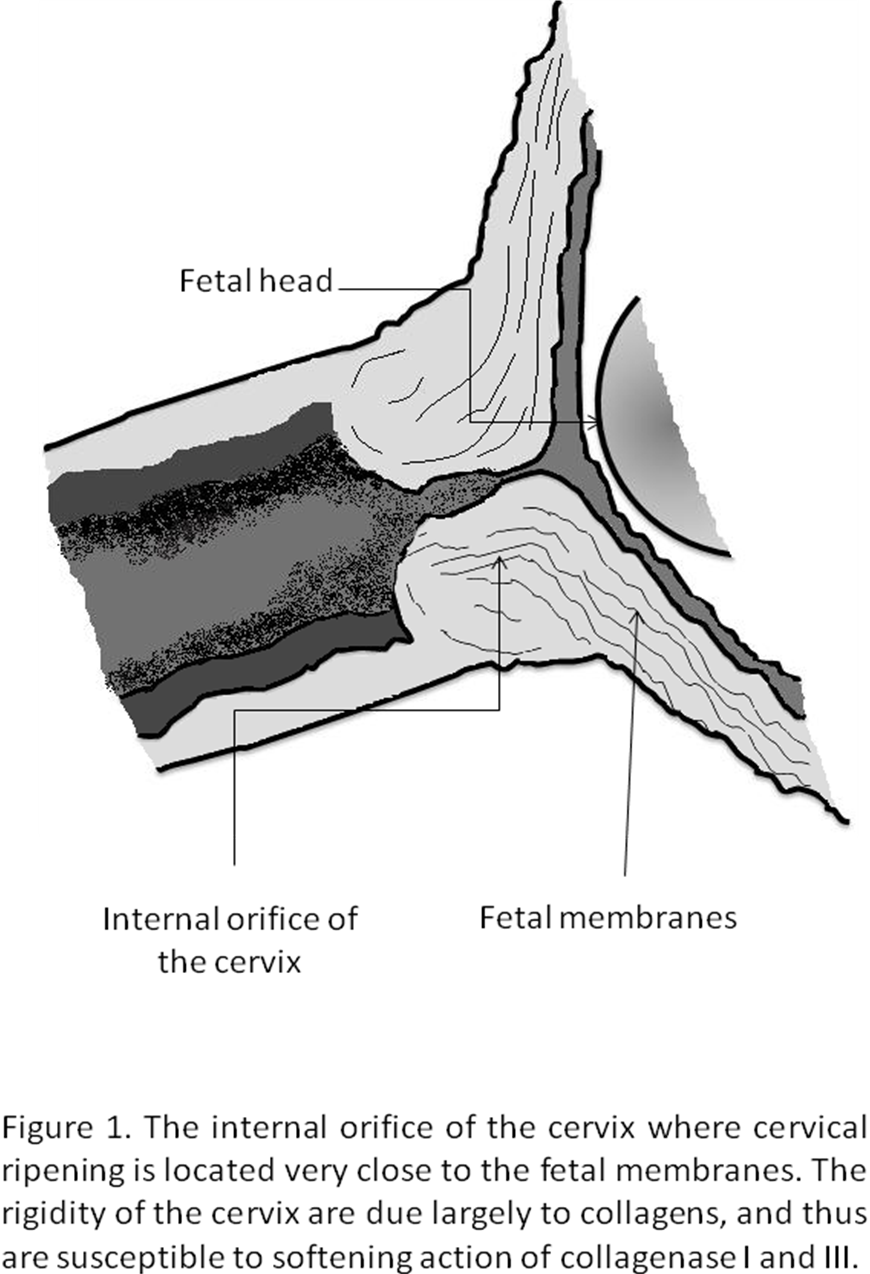


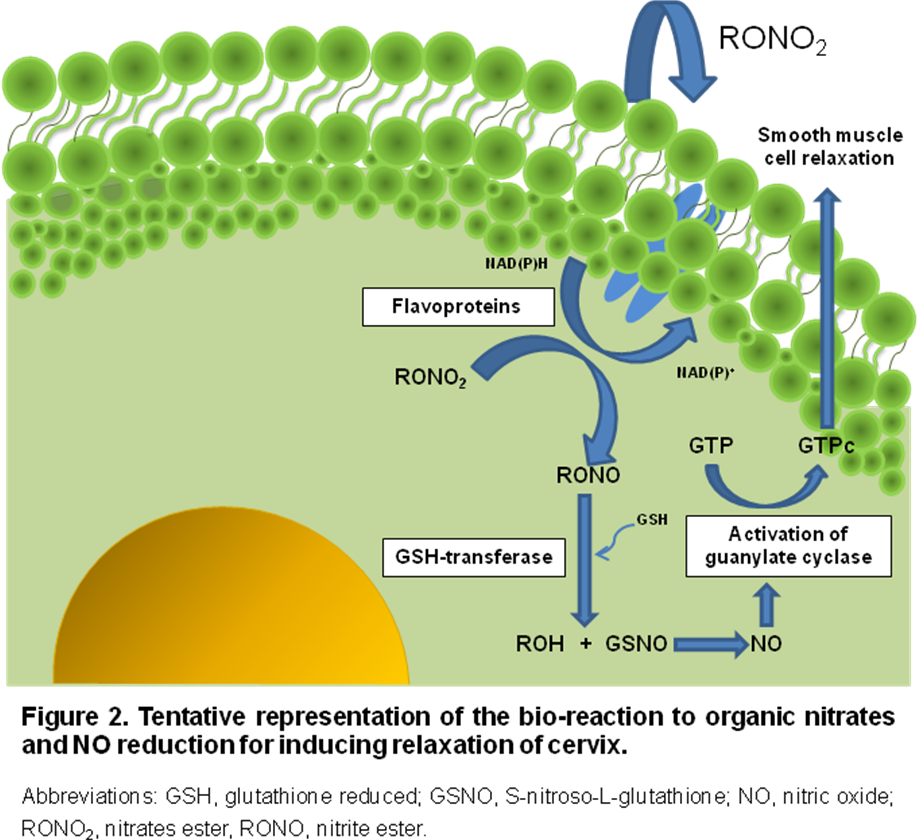


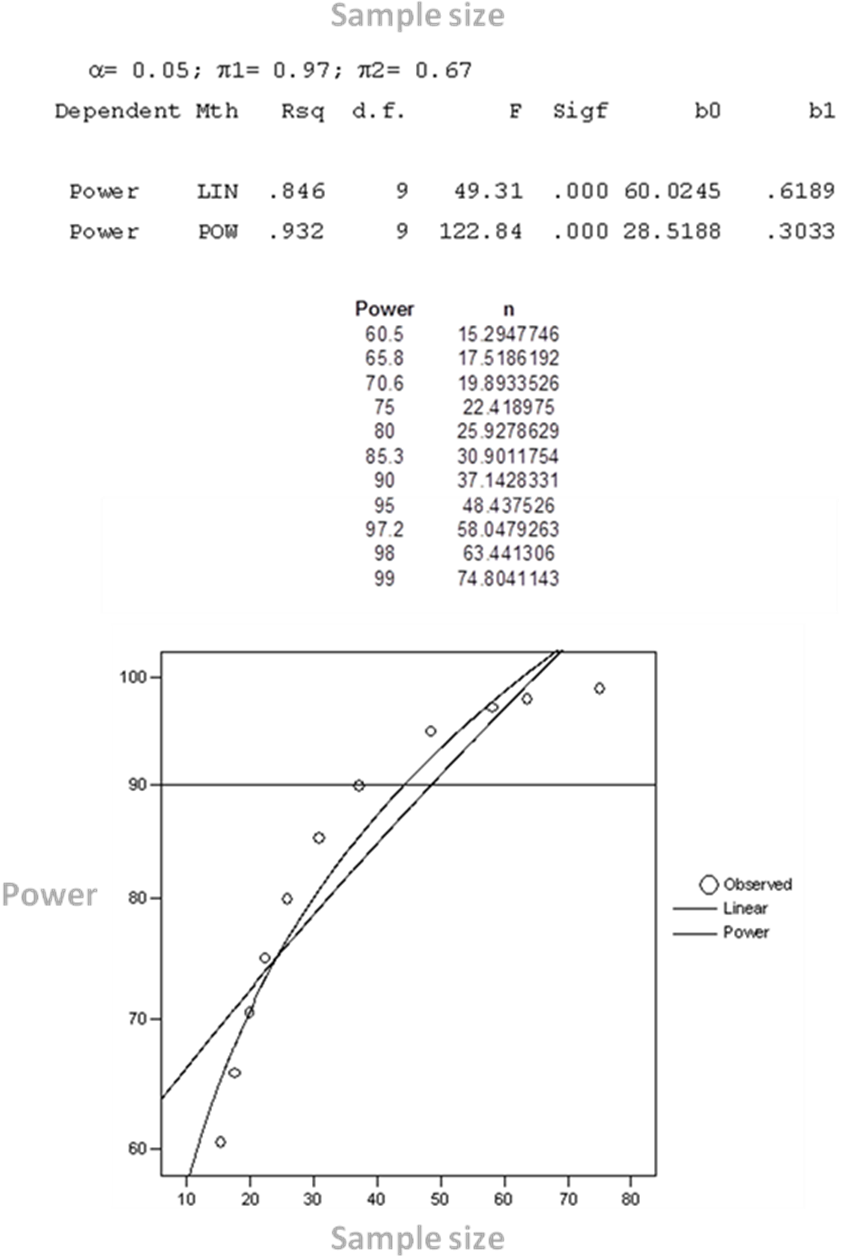

Supplement: S1 Protocol — (DOC) [file pone.0215718.s003.doc]
